# The pMSSM10 after LHC run 1

**DOI:** 10.1140/epjc/s10052-015-3599-y

**Published:** 2015-09-15

**Authors:** K. J. de Vries, E. A. Bagnaschi, O. Buchmueller, R. Cavanaugh, M. Citron, A. De Roeck, M. J. Dolan, J. R. Ellis, H. Flächer, S. Heinemeyer, G. Isidori, S. Malik, J. Marrouche, D. Martínez Santos, K. A. Olive, K. Sakurai, G. Weiglein

**Affiliations:** High Energy Physics Group, Blackett Laboratory, Imperial College, Prince Consort Road, London, SW7 2AZ UK; DESY, Notkestraße 85, 22607 Hamburg, Germany; Fermi National Accelerator Laboratory, P.O. Box 500, Batavia, IL 60510 USA; Physics Department, University of Illinois at Chicago, Chicago, IL 60607-7059 USA; Physics Department, CERN, 1211 Geneva 23, Switzerland; Antwerp University, 2610 Wilrijk, Belgium; Theory Group, SLAC National Accelerator Laboratory, 2575 Sand Hill Road, Menlo Park, CA 94025-7090 USA; ARC Centre of Excellence for Particle Physics at the Terascale, School of Physics, University of Melbourne, Melbourne, 3010 Australia; Theoretical Particle Physics and Cosmology Group, Department of Physics, King’s College London, London, WC2R 2LS UK; H.H. Wills Physics Laboratory, University of Bristol, Tyndall Avenue, Bristol, BS8 1TL UK; Instituto de Física de Cantabria (CSIC-UC), 39005 Santander, Spain; Physik-Institut, Universität Zürich, 8057 Zurich, Switzerland; Nikhef National Institute for Subatomic Physics, VU University Amsterdam, Amsterdam, The Netherlands; Universidade de Santiago de Compostela, 15706 Santiago de Compostela, Spain; William I. Fine Theoretical Physics Institute, School of Physics and Astronomy, University of Minnesota, Minneapolis, MN 55455 USA

## Abstract

We present a frequentist analysis of the parameter space of the pMSSM10, in which the following ten soft SUSY-breaking parameters are specified independently at the mean scalar top mass scale $M_\mathrm{SUSY}\equiv \sqrt{m_{\tilde{t}_{1}} m_{\tilde{t}_{2}}}$: the gaugino masses $M_{1,2,3}$, the first-and second-generation squark masses $m_{\tilde{q}_1}= m_{\tilde{q}_2}$, the third-generation squark mass $m_{\tilde{q}_3}$, a common slepton mass $m_{\tilde{\ell }}$ and a common trilinear mixing parameter *A*, as well as the Higgs mixing parameter $\mu$, the pseudoscalar Higgs mass $M_A$ and $\tan \beta$, the ratio of the two Higgs vacuum expectation values. We use the MultiNest sampling algorithm with $\sim$1.2 $\times 10^9$ points to sample the pMSSM10 parameter space. A dedicated study shows that the sensitivities to strongly interacting sparticle masses of ATLAS and CMS searches for jets, leptons $+$ signals depend only weakly on many of the other pMSSM10 parameters. With the aid of the Atom and Scorpion codes, we also implement the LHC searches for electroweakly interacting sparticles and light stops, so as to confront the pMSSM10 parameter space with all relevant SUSY searches. In addition, our analysis includes Higgs mass and rate measurements using the HiggsSignals code, SUSY Higgs exclusion bounds, the measurements of $\mathrm{BR}(B_s \rightarrow \mu ^+\mu ^-)$ by LHCb and CMS, other *B*-physics observables, electroweak precision observables, the cold dark matter density and the XENON100 and LUX searches for spin-independent dark matter scattering, assuming that the cold dark matter is mainly provided by the lightest neutralino $\tilde{\chi }^0_{1}$. We show that the pMSSM10 is able to provide a supersymmetric interpretation of $(g-2)_\mu$, unlike the CMSSM, NUHM1 and NUHM2. As a result, we find (omitting Higgs rates) that the minimum $\chi ^2 = 20.5$ with 18 degrees of freedom (d.o.f.) in the pMSSM10, corresponding to a $\chi ^2$ probability of 30.8 %, to be compared with $\chi ^2/\mathrm{d.o.f.} = 32.8/24 \ (31.1/23) \ (30.3/22)$ in the CMSSM (NUHM1) (NUHM2). We display the one-dimensional likelihood functions for sparticle masses, and we show that they may be significantly lighter in the pMSSM10 than in the other models, e.g., the gluino may be as light as $\sim$1250 $\,\, \mathrm {GeV}$ at the 68 % CL, and squarks, stops, electroweak gauginos and sleptons may be much lighter than in the CMSSM, NUHM1 and NUHM2. We discuss the discovery potential of future LHC runs, $e^+e^-$ colliders and direct detection experiments.

## Introduction

The quest for supersymmetry (SUSY) has been among the principal objectives of the ATLAS and CMS experiments during run 1 of the Large Hadron Collider (LHC). However, despite searches in many production and decay channels, no significant signals have been observed [1–4]. These negative results impose strong constraints on *R*-conserving SUSY models, in particular, which are also constrained by measurements of the mass and other properties of the Higgs boson [5, 6], by precision measurements of rare decays such as $B_s \rightarrow \mu ^+ \mu ^-$ [7–10] and other measurements. Overall, these constraints tend to reduce the capacity of SUSY models to alleviate the hierarchy problem. However, their impact on a possible resolution of the discrepancy between the experimental measurement of $(g-2)_\mu$ and theoretical calculations in the Standard Model (SM) depends on further assumptions as will be discussed below.

There have been many analyses that combine these constraints in global statistical fits within specific SUSY models based on the minimal supersymmetric extension of the Standard Model (MSSM) [11, 12]. Many of these analyses assume that the low-energy soft SUSY-breaking parameters of the MSSM may be extrapolated using the renormalisation-group equations (RGEs) up to some grand unified theory (GUT) scale, where they are postulated to satisfy some universality conditions. Examples of such models include the constrained MSSM (CMSSM) [13–30], in which the soft SUSY-breaking mass parameters $m_0$ and $m_{1/2}$ are assumed to be universal at the GUT scale, as are the trilinear parameters $A_0$. Other examples include models that relax the universality assumptions for the soft SUSY-breaking contributions to the Higgs masses, the NUHM1 [31–34] and NUHM2 [35, 36] (see also, e.g., Ref. [30]), but retain universality for the slepton, squark and gaugino masses. Such models are particularly severely constrained by the LHC searches for colored sparticles, the squarks and gluino, which also place indirect limits on the masses of sleptons and electroweak gauginos and higgsinos via the GUT-scale constraints, while the direct search limits on these particles have much less impact.

An alternative approach is to make no assumption concerning the RGE extrapolation to very high energies, but take a purely phenomenological approach in which the soft SUSY-breaking parameters are specified at low energies and are not required to be universal at any input scale, a class of models referred to as the phenomenological MSSM with *n* free parameters (pMSSM*n*) [37–50]. This is the framework explored in this paper. Favoured mass patterns in a pMSSM*n* analysis might then give hints for (alternative) GUT-scale scenarios.

In the absence of any assumptions, the pMSSM has so many parameters that a thorough analysis of its multi-dimensional parameter space is computationally prohibitive. Here we restrict our attention to a ten-dimensional version, the pMSSM10, in which the following assumptions are made. Motivated by the absence of significant flavour-changing neutral interactions (FCNI) beyond those in the Standard Model (SM), we assume that the soft SUSY-breaking contributions to the masses of the squarks of the first two generations are equal, which we also assume for the three generations of sleptons. The FCNI argument does not motivate any relation between the soft SUSY-breaking contributions to the masses of left- and right-handed sfermions, but here we assume for simplicity that they are equal. As a result, we consider the following ten parameters in our analysis (where “mass” is here used as a synonym for a soft SUSY-breaking parameter, and the gaugino masses and trilinear couplings are taken to be real):

1 $$\begin{aligned} \mathrm{3~gaugino~masses:}&\; M_{1,2,3}, \nonumber \\ \mathrm{2~squark~masses:}&\; m_{\tilde{q}_1} \, = \, m_{\tilde{q}_2} \, \ne \, m_{\tilde{q}_{3}}, \nonumber \\ \mathrm{1~slepton~mass:}&\; m_{\tilde{\ell }}, \nonumber \\ \mathrm{1~trilinear~coupling:}&\; A , \\ \mathrm{Higgs~mixing~parameter:}&\; \mu , \nonumber \\ \mathrm{Pseudoscalar~Higgs~mass:}&\; M_A, \nonumber \\ \mathrm{Ratio~of~vevs:}&\; \tan \beta . \nonumber \end{aligned}$$

All of these parameters are specified at a low renormalisation scale, the mean scalar top mass scale, $M_\mathrm{SUSY}\equiv \sqrt{m_{\tilde{t}_{1}} m_{\tilde{t}_{2}}}$, close to that of electroweak symmetry breaking.

In any pMSSM scenario such as this, the disconnect between the different gaugino masses allows, for example, the U(1) and SU(2) gauginos to be much lighter than is possible in GUT-universal models, where their masses are related to the gluino mass and hence constrained by gluino searches at the LHC. Likewise, the disconnect between the different squark masses opens up more possibilities for light stops, and the disconnect between squark and slepton masses largely frees the latter from LHC constraints.

An important feature of our global analysis is that the possibilities for light electroweak gauginos and sleptons reopen an opportunity for an significant SUSY contribution to $(g-2)_\mu$ in the pMSSM, a possibility that is precluded in simple GUT-universal models such as the CMSSM, NUHM1 and NUHM2 by the LHC searches for strongly interacting sparticles. As we discuss in detail in this paper, the pMSSM10 flexibility removes the tension between LHC constraints and the measured value of $(g-2)_\mu$[51, 52], with the result that the best fit in the pMSSM10 has a global $\chi ^2$ probability that is considerably better than in the CMSSM, NUHM1, NUHM2 or SM.

The main challenges for a global fit of the pMSSM10 are the efficient sampling of the ten-dimensional parameter space and the accurate implementation of the various SUSY searches by ATLAS and CMS. As in [53], here we use the sampling algorithm MultiNest [54–56] to scan efficiently the pMSSM10 parameter space. To achieve sufficient coverage of the relevant parameter space, approximately $1.2 \times 10^9$ pMSSM10 points were sampled. However, confronting all these sample points individually with all relevant collider searches is computationally impossible. In order to overcome this problem and still to apply the SUSY searches in a consistent and precise manner, we split the LHC searches into three categories. In the first category we consider inclusive SUSY searches that mainly constrain the production of coloured sparticles, namely the gluino and squarks. To apply these searches to the pMSSM10 parameter space, we follow closely an approach proposed in [57], which uses a variety of inclusive SUSY searches covering different final states to establish a simple but accurate look-up table that depends only on the gluino, squark and LSP masses. Then, in order to implement the other two categories of LHC constraints on the SUSY electroweak sector and compressed stop spectra, we treat the LHC searches for electroweakly interacting sparticles via trileptons and dileptons, and for light stops, separately using dedicated algorithms validated using the Atom [58] and Scorpion^1^ codes. In all cases we consider the latest SUSY searches from ATLAS and CMS that are based on the full run 1 data set, as detailed later in the paper. We perform extensive validations of the applications of these searches to the pMSSM10, so as to ensure that we make an accurate and comprehensive set of implementations of the experimental constraints on the model.

More information as regards the scan of the pMSSM10 parameter space using the MultiNest technique, as well as details as regards our implementations of the LHC searches, are provided in Sect. 2. Section 3 discusses the results of the pMSSM10 analysis, including the best-fit point and other benchmark points with low sparticle masses that could serve to focus analyses at run 2 of the LHC. Section 4 discusses the extent to which the preferred ranges of pMSSM10 parameters permit renormalisation-group extrapolation to GUT scales. Section 5 analyses the prospects for discovering SUSY in future runs of the LHC, Sect. 6 analyses the prospects for discovering SUSY at possible future $e^+ e^-$ colliders, and our conclusions are summarised in Sect. 7.

## Method

We describe in this section how we perform a global fit of the pMSSM10 taking into account constraints from direct searches for SUSY particles, the Higgs boson mass and rate measurements, SUSY Higgs exclusion bounds, precision electroweak observables, *B*-physics observables, and astrophysical and cosmological constraints on cold dark matter. We describe the scanned parameters and their ranges, the framework that we use to calculate the observables, and the treatment of the various constraints.

### Parameter ranges

As described above we consider a ten-dimension subset (pMSSM10) of the full pMSSM parameter space. The selected SUSY parameters were listed in Eq. (1), and the ranges of these parameters that we sample are shown in Table 1. We also indicate in the right column of this table how we divide the ranges of most of these parameters into segments, as we did previously for our analyses of the CMSSM, NUHM1 and NUHM2 [53, 59].

The combinations of these segments constitute boxes, in which we sample the parameter space using the MultiNest package [54]. For each box, we choose a prior for which 80 % of the sample has a flat distribution within the nominal range, and 20 % of the sample is outside the box in normally distributed tails in each variable. In this way, our total sample exhibits a smooth overlap between boxes, eliminating features associated with box boundaries. An initial scan over all mass parameters with absolute values $\le$4000$\,\, \mathrm {GeV}$showed that non-trivial behaviour of the global likelihood function was restricted to $|M_1|\lesssim 500\,\, \mathrm {GeV}$ and $m_{\tilde{l}}\lesssim 1000\,\, \mathrm {GeV}$. In order to achieve high resolution efficiently, we restricted the ranges of these parameters to $|M_1|<1000\,\, \mathrm {GeV}$ and $0<m_{\tilde{l}}<2000\,\, \mathrm {GeV}$ in the full scan.

**Table 1:** Ranges of the pMSSM10 parameters sampled, together with the numbers of segments into which each range was divided, and the corresponding number of sample boxes

Parameter	Range	Number of segments
$M_1$ (TeV)	$(-1 , 1 )$	2
$M_2$ (TeV)	( 0 , 4 )	2
$M_3$ (TeV)	$(-4 , 4 )$	4
$m_{\tilde{q}}$ (TeV)	( 0 , 4 )	2
$m_{\tilde{q}_3}$ (TeV)	( 0 , 4 )	2
$m_{\tilde{l}}$ (TeV)	( 0 , 2 )	1
$M_A$ (TeV)	( 0 , 4 )	2
*A* (TeV)	$(-5 , 5 )$	1
$\mu$ (TeV)	$(-5 , 5 )$	1
$\tan \beta$	( 1 , 60)	1
Total number of boxes		128

### MasterCode framework

We calculate the observables that go into the likelihood using the MasterCode framework [53, 59–63], which interfaces various public and private codes: SoftSusy 3.3.9 [64] for the spectrum, FeynWZ [65, 66] for the electroweak precision observables, FeynHiggs 2.10.0 [67–71] for the Higgs sector and $(g-2)_\mu$, SuFla [72, 73], SuperIso [74–76] for the *B*-physics observables, Micromegas 3.2 [77–79] for the dark matter relic density, SSARD^2^ for the spin-independent cross-section $\sigma ^\mathrm{SI}_p$, SDECAY 1.3b [80] for calculating sparticle branching ratios, and HiggsSignals1.3.0 [81, 82] and HiggsBounds 4.2.0 [83–85] for calculating constraints on the Higgs sector. The codes are linked using the SUSY Les Houches Accord (SLHA) [86, 87].

### Electroweak, flavour, cosmological and dark matter constraints

For many of these constraints, we follow very closely our previous implementations, which were summarised recently in Table 1 in [53]. Specifically, we treat all electroweak precision observables, all *B*-physics observables (except for $\mathrm{BR}(B_{s, d} \rightarrow \mu ^+\mu ^-)$), $(g-2)_\mu$ and the relic density as Gaussian constraints. The $\chi ^2$ contribution from $\mathrm{BR}(B_{s, d} \rightarrow \mu ^+\mu ^-)$, combined here in the quantity $R_{\mu \mu }$ [59], is calculated using the combination of CMS [8] and LHCb [7] results described in [10]. We incorporate the current world average of the branching ratio for BR($b \rightarrow s \gamma$) from [88] combined with the theoretical estimate in the SM from [89], and the recent measurement of the branching ratio for BR($B_u \rightarrow \tau \nu _\tau$) by the Belle Collaboration [90] combined with the SM estimate from [91]. We use the upper limit on the spin-independent cross section as a function of the lightest neutralino mass $m_{\tilde{\chi }^0_{1}}$ from LUX [92], which is slightly stronger than that from XENON100 [93], taking into account the theoretical uncertainty on $\sigma ^\mathrm{SI}_p$ as described in [59].

### Higgs constraints

We use the recent combination of ATLAS and CMS measurements of the mass of the Higgs boson: $M_h= 125.09 \pm 0.24 \,\, \mathrm {GeV}$ [94], which we combine with a one-$\sigma$ uncertainty of $1.5 \,\, \mathrm {GeV}$ in the FeynHiggs calculation of $M_h$ in the MSSM.

In addition, we refine substantially our treatment of the Higgs boson constraints, as compared with previous analyses in the MasterCode framework. In order to include the observed Higgs signal rates we have incorporated HiggsSignals [81, 82], which evaluates the $\chi ^2$ contribution of 77 channels from the Higgs boson searches at the LHC and the Tevatron (see Ref. [81, 82] for a complete list of references). A discussion of the effective number of contributing channels is given in Sect. 3.2 below.

We also take into account the relevant searches for heavy neutral MSSM Higgs bosons via the $H/A \rightarrow \tau ^+\tau ^-$ channels [95, 96]. We evaluate the corresponding $\chi ^2$ contribution using the code HiggsBounds [83–85], which includes the latest CMS results [95] based on $\sim 25~\mathrm{fb}^{-1}$ of data.^3^ These results include a combination of the two possible production modes, $gg \rightarrow H/A$ and $b \bar{b} \rightarrow b \bar{b} H/A$, which is consistently evaluated depending on the MSSM parameters. Their implementation in HiggsBounds has been tested against the published CMS data, and very good qualitative and quantitative agreement had been found [97]. Other Higgs boson searches are not taken into account, as they turn out to be weaker in the pMSSM10 that we study.

### LHC constraints on sparticle masses

A comprehensive and accurate application of the SUSY searches with the full run 1 data of the LHC to the pMSSM10 parameter space is a central part of this paper. As most of these searches have been interpreted by ATLAS and CMS only in simplified model frameworks, we have introduced supplementary procedures in order to apply these searches to the complicated sparticle spectrum content of a full SUSY model such as the pMSSM10. For this we consider three separate categories of particle mass constraints that arise from the LHC searches: (a) generic constraints on coloured sparticles (gluinos and squarks), (b) dedicated constraints on electroweakly interacting gauginos, Higgsinos and sleptons, (c) dedicated constraints on stop production in scenarios with compressed spectra. We refer to the combination of all these constraints from direct SUSY searches as the LHC8 constraint, with sectors labelled as $\mathrm{LHC8}_\mathrm{col}$, $\mathrm{LHC8}_\mathrm{EWK}$ and $\mathrm{LHC8}_\mathrm{stop}$, respectively. In the following subsections we provide further details as regards our implementations of these individual constraints, discussing in detail the validations of our procedures and the corresponding uncertainties.

We use two dedicated software frameworks for recasting the LHC analyses used in this paper. Both frameworks implement the full list of cuts of a given experimental search to obtain yields in the respective signal regions of the search. These signal yields are then confronted with the SM background yields and observations in data, as reported by the experimental searches. Based on these comparisons we construct the standard statistical estimator $\mathrm{CL}_s$ [98], which is also used by the experiments to determine the compatibility of their data with a given signal hypothesis. In this way it is possible to interpret the various LHC searches in any given SUSY model, such as those explored in our pMSSM10 scans.

To recast the ATLAS searches considered in this paper we use Atom [58], which is a Rivet [99] based framework. Atom models the resolutions of LHC detectors by mapping from the truth-level particles found for example in PYTHIA 6 [100] event samples to the reconstructed objects, such as *b*-jets and isolated leptons, according to the reported detector performances. In particular, the efficiencies of object reconstruction and the parameters associated with the momentum smearing are implemented in the form of analytical functions or numerical grids. The program has already been used in several studies [101–104], and the validation of the code can be found in [105].

For the CMS searches we use a private code called Scorpion^4^ that was already used in [57]. Scorpion obtains signal yields for a number of CMS searches based on events generated with PYTHIA 6 [100] that are passed through the DELPHES 3 [106, 107] detector simulation package using an appropriate data card to emulate the response of the CMS detector. A significant effort was made to validate the modelling of these analyses by comparing the results obtained with the published results of the experimental collaboration. For further information on the validation of the CMS searches see [57].

The signal yields from Atom and Scorpion are confronted with the background yields and observations obtained from the individual ATLAS and CMS searches, and the corresponding $\mathrm{CL}_s$ is calculated using the LandS package [108]. We convert the calculated $\mathrm{CL}_s$ value for a generic spectrum in the MSSM into a $\chi ^2$ contribution by interpreting it as a *p* value for the signal hypothesis assuming one degree of freedom.

#### LHC constraints on coloured sparticles

In the cases of the CMSSM, NUHM1 and NUHM2, we showed in [53, 59] that it was sufficient to extrapolate to other parameter values the exclusion contour in the CMSSM $(m_0,m_{1/2})$ plane from the ATLAS search for jets$+$ [1, 2] that was given for specified values of $\tan \beta$ and $A_0$. We showed that the ATLAS exclusion is, to good approximation, independent of $\tan \beta$ and $A_0$ [53, 61, 109] and, for the applications to the NUHM1 and the NUHM2, we checked that these limits in the $(m_0,m_{1/2})$ plane were independent of the degrees of non-universality of the soft SUSY-breaking contributions to the Higgs masses, within the intrinsic sampling uncertainties.

In the case of the pMSSM10, however, the implementation of the direct searches for coloured sparticles is less straightforward. It is computationally impossible to apply all the LHC search constraints individually to each of the $$\sim $$$$1.2 \times 10^9$$ parameter choices in our sample. For example, PYTHIA 6 and DELPHES 3 take several minutes for the generation of 10 000 events followed by detector simulation, which is required to determine the signal acceptance and $$\mathrm{CL}_s$$ of each point sampled in the parameter space. Instead, we follow an approach outlined in [57], which constructs universal mass limits on coloured sparticles by combining an inclusive set of jets $$+$$*X*$$+$$ searches, as we now describe.

As was shown in [57], it is possible to establish lower limits on the gluino mass, $m_{\tilde{g}}$, and the third-generation squark mass, $m_{\tilde{q}_3}$, that are independent of the details of the underlying spectrum, within the intrinsic sampling uncertainties, by combining a suitable set of inclusive SUSY searches. In this approach the limits only depend on $m_{\tilde{g}}$, $m_{\tilde{q}_3}$ and the mass of the lightest sparticle $m_{\tilde{\chi }^0_{1}}$. The essence of the idea is that strongly interacting sparticles decay through a variety of different cascade channels, whose relative probabilities depend on other model parameters. However, if one combines a sufficiently complete set of channels of the form jets $+$*X*$+$, one will capture essentially all the relevant decay channels.

In order to apply this idea to the pMSSM10 parameter space, we have to extend this approach to include also the generic first- and second-generation squark mass, $m_{\tilde{q}}$, as a free parameter. We then construct a ‘universal’ $\chi ^2$ function that depends only on $m_{\tilde{\chi }^0_{1}}$, $m_{\tilde{g}}$, $m_{\tilde{q}}$ and $m_{\tilde{q}_3}$, as detailed below. This function defines our implementation of this $\mathrm{LHC8}_\mathrm{col}$ constraint. There are two caveats to this approach. One is that the region of parameter space where $m_{\tilde{t}_{1}} - m_{\tilde{\chi }^0_{1}}$ is small, which is the object of dedicated searches, requires special attention. The other is that searches for electroweakly produced sparticles (sleptons, neutralinos and charginos) fall outside the scope of the $\mathrm{LHC8}_\mathrm{col}$ constraint. We have developed dedicated approaches to establishing accurate LHC limits for the special cases of electroweakly produced sparticles and the compressed-stop scenario with $m_{\tilde{t}_{1}} - m_{\tilde{\chi }^0_{1}} < m_t$, as described in Sects. 2.5.2 and 2.5.3, respectively.

In order to construct $\chi ^2$ as a function of $m_{\tilde{\chi }^0_{1}}$, $m_{\tilde{g}}$, $m_{\tilde{q}}$ and $m_{\tilde{q}_3}$, we first generate a sample of points on a $1+3$ dimensional grid, which we use for linear interpolation. We construct this grid starting from values of $m_{\tilde{\chi }^0_{1}} = \{10,~110,~\ldots ,~610\}~\,\, \mathrm {GeV}$. For each of these values of $m_{\tilde{\chi }^0_{1}}$, we select the following values of $m_{\tilde{g}}$ and $m_{\tilde{q}}$: $\{m_{\tilde{\chi }^0_{1}}+40,~m_{\tilde{\chi }^0_{1}}+140,~\ldots ,~1750,~2500,~5000\} \,\, \mathrm {GeV}$, whereas $m_{\tilde{q}_3}$ takes values $\{m_{\tilde{\chi }^0_{1}}+80,~m_{\tilde{\chi }^0_{1}}+180,~\ldots , 1290,~2500,~5000\}~\,\, \mathrm {GeV}$, where the dots indicate steps of 100 $\,\, \mathrm {GeV}$, so that the total number of points in the grid is 25,564. The choice for this grid is motivated by the need for a fine granularity at low masses, while also capturing the parameter behaviours at higher masses.

We associate a SUSY spectrum to each point on the grid, by setting the first- and second-generation squark masses equal to $m_{\tilde{q}}$, and the third-generation squark masses equal to $m_{\tilde{q}_3}$. For each SUSY spectrum we generate coloured sparticle production events using PYTHIA 6 [100] and pass them through the DELPHES 3 [106, 107] detector simulation code using a detector card that emulates the CMS detector response. We then pass the resulting events through Scorpion,^5^ which emulates the monojet, MT2, single-lepton, same- and opposite-sign dilepton (SS and OS) and 3-lepton CMS searches [110–115], to estimate the numbers of signal events in each of the signal regions. After this we calculate the $\mathrm{CL}_s$ using the LandS package [108], by combining all signal regions from these searches. If searches have overlapping signal regions, we take the strongest expected limit, as is the case for the CMS monojet and single-lepton searches.

**Fig. 1:**
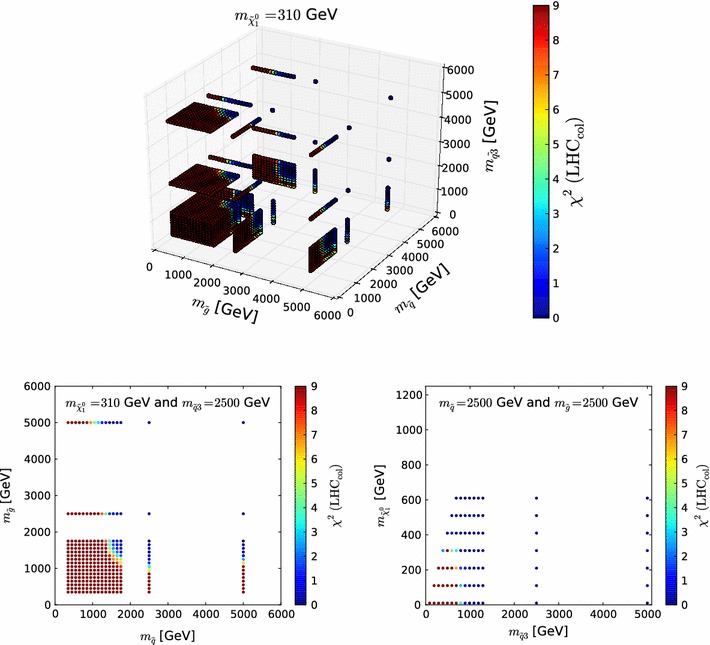
Illustration of the grid in $m_{\tilde{\chi }^0_{1}}$, $m_{\tilde{g}}$, $m_{\tilde{q}}$ and $m_{\tilde{q}_3}$ on which $\chi ^2(\mathtt{Scorpion})$ is evaluated in order to construct $\mathrm{LHC8}_\mathrm{col}$. The *upper panel* shows the three-dimensional grid for $m_{\tilde{\chi }^0_{1}}=310\,\, \mathrm {GeV}$, the *lower left panel* shows a two-dimensional slice through the grid, and the *lower right panel* is another two-dimensional slice that illustrates the dependence on $m_{\tilde{\chi }^0_{1}}$; see the text

In Fig. 1 we show a three-dimensional overview and a pair of two-dimensional slices through this grid. The top panel shows the full three-dimensional grid for $m_{\tilde{\chi }^0_{1}}=310\,\, \mathrm {GeV}$ and illustrates the fine and coarse granularity of the grid at low and high values of $m_{\tilde{g}}$  $m_{\tilde{q}}$ and $m_{\tilde{q}_3}$, respectively. The lower left panel shows the two-dimensional slice for the same neutralino mass and $m_{\tilde{q}_3}=2500\,\, \mathrm {GeV}$, highlighting that there is only a small, though non-negligible, dependence of the $\chi ^2$ function on $m_{\tilde{q}}$ for values of $m_{\tilde{g}}\gtrsim 2500\,\, \mathrm {GeV}$. The lower right panel shows the $\chi ^2$ function as a function of $m_{\tilde{q}_3}$ and $m_{\tilde{\chi }^0_{1}}$, for fixed $m_{\tilde{q}}=2500\,\, \mathrm {GeV}$ and $m_{\tilde{g}}=2500\,\, \mathrm {GeV}$, illustrating that for different values of $m_{\tilde{\chi }^0_{1}}$ different grids are defined in $m_{\tilde{g}}$, $m_{\tilde{q}}$ and $m_{\tilde{q}_3}$.

**Fig. 2:**
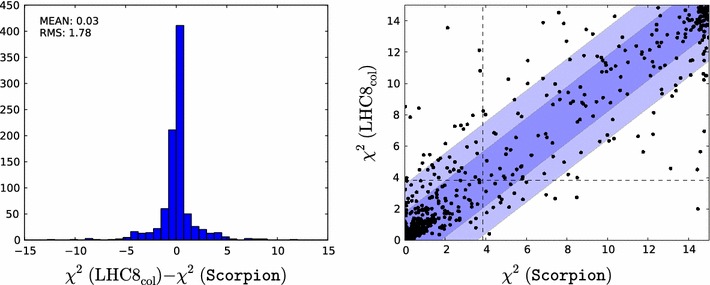
*Left panel* histogram of the differences between the values of the likelihood function ${\chi ^2(\mathtt{Scorpion})}$ evaluated using individual $\mathrm{LHC8}_\mathrm{col}$ searches for 1000 randomly selected points and the estimate ${\chi ^2(\mathrm{LHC8}_\mathrm{col})}$ obtained by interpolation from a look-up table as described in the text. *Right panel* scatter plot in the $({\chi ^2(\mathtt{Scorpion})}, {\chi ^2(\mathrm{LHC8}_\mathrm{col})})$ plane of the $\chi ^2$ values obtained from the two approaches; the *vertical and horizontal dashed lines* in this plot correspond to the 95 % $\mathrm{CL}_s$ in each approach

In order to apply the $\mathrm{LHC8}_\mathrm{col}$ constraint to a generic pMSSM10 spectrum, we calculate $m_{\tilde{q}}$ ($m_{\tilde{q}_3}$) as the cross-section-weighted average of the first- and second- (third-) generation squark masses, to ensure that the $\mathrm{LHC8}_\mathrm{col}$ constraint reflects the actual production cross sections. This is especially relevant for the third-generation squark masses, as they generally have large splittings. The $\chi ^2$ contribution for $\mathrm{LHC8}_\mathrm{col}$ is obtained by linear interpolation of the $\chi ^2$ values on the $1+3$-dimensional grid. There is one special case when $m_{\tilde{t}_{1}}-m_{\tilde{\chi }^0_{1}} < m_t$: here the standard searches listed above are less sensitive, and the universality of the limits is expected to break down. In this case, we calculate $m_{\tilde{q}_3}$ assuming zero cross section for the lighter stop, and we consider separately the impacts of dedicated stop searches in this region, as described in Sect. 2.5.3.

In order to validate the $\mathrm{LHC8}_\mathrm{col}$ constraint and to gauge quantitatively its uncertainty, we have performed a number of studies and tests. First, we randomly selected 1000 model points from our sample where at least one of the sparticle masses is low enough to have been within the reach of LHC run 1 ($m_{\tilde{\chi }^0_{1}}<600 \,\, \mathrm {GeV}$ and either $m_{\tilde{g}}<1500 \,\, \mathrm {GeV}$, $m_{\tilde{q}}<1600 \,\, \mathrm {GeV}$ or $m_{\tilde{q}_3}<900 \,\, \mathrm {GeV}$) and $\Delta \chi ^2 < 10$ relative to the global minimum. For these points we compare the $\chi ^2$ values interpolated from the look-up table (${\chi ^2(\mathrm{LHC8}_\mathrm{col})}$) with the $\chi ^2$ obtained by running the full chain of event generation, detector simulation and analyses (${\chi ^2(\mathtt{Scorpion})}$). The left panel of Fig. 2 shows a histogram of the differences for the 1000 randomly selected points. As indicated in the legend of this figure, the standard deviation on this distribution is $\sigma _{\chi ^2}=1.8$.

The right panel of Fig. 2 shows a scatter plot in the $$({\chi ^2(\mathtt{Scorpion})}, {\chi ^2(\mathrm{LHC8}_\mathrm{col})})$$ plane of the $$\chi ^2$$ values obtained from the two approaches. They would agree perfectly along the diagonal where $${\chi ^2(\mathtt{Scorpion})}= {\chi ^2(\mathrm{LHC8}_\mathrm{col})}$$, and the lighter- and darker-shaded blue strips are the $$\pm 1 \sigma _{\chi ^2}$$ and $$\pm 2 \sigma _{\chi ^2}$$ bands around this diagonal. The vertical and horizontal dashed lines in this plot correspond to the 95 % $$\mathrm{CL}_s$$ in each approach. For the majority of points, the interpolation and the full analysis agree whether the point is excluded at the 95 % $$\mathrm{CL}_s$$, or not, and most of the remaining points lie within $$\pm $$$$2 \sigma _{\chi ^2}$$.

**Fig. 3:**
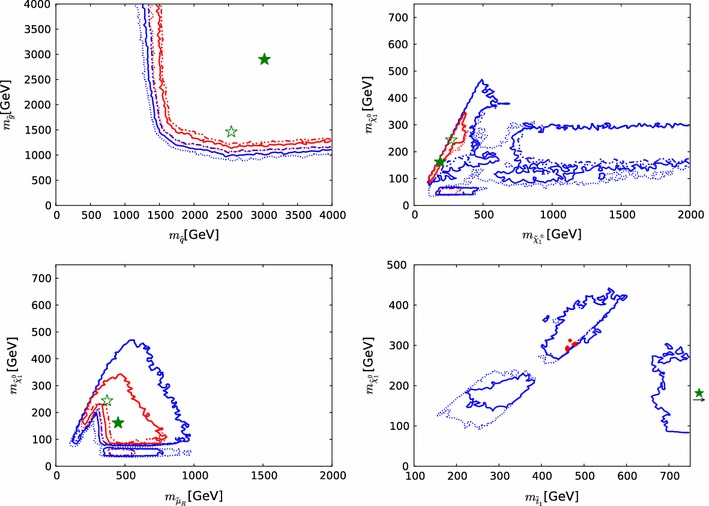
Impacts of the $\pm$1 $\sigma$ uncertainties in our implementations of the $\mathrm{LHC8}_\mathrm{col}$, $\mathrm{LHC8}_\mathrm{EWK}$ and $\mathrm{LHC8}_\mathrm{stop}$ constraints on the 68 and 95 % CL regions (indicated by the *red* and *blue contours*) in the corresponding relevant mass planes: $(m_{\tilde{q}}, m_{\tilde{g}})$ (*upper left panel*), $(m_{\tilde{\chi }^\pm _{1}}, m_{\tilde{\chi }^0_{1}})$ (*upper right panel*), $(\tilde{\mu }_{R}, m_{\tilde{\chi }^0_{1}})$ (*lower left panel*), and $(m_{\tilde{t}_{1}}, m_{\tilde{\chi }^0_{1}})$ (*lower right panel*). In each case, the *dot-dashed* and *dashed contours* are obtained by shifting the respective $\chi ^2$ penalty up and down by one standard deviation $\sigma _{\chi ^2}$, as discussed in the text. The *filled green stars* correspond to the nominal best-fit point and the *open stars* (shown if not overlapping) to those which were obtained from shifting the $\chi ^2$ up or down with $\sigma _{\chi ^2}$. We note that in the *lower right panel* the best-fit points lie outside the displayed parameter range

We then assess how the uncertainty $\sigma _{\chi ^2}$ in our implementation of the $\mathrm{LHC8}_\mathrm{col}$ constraint translates into uncertainties in sparticle mass limits: see the upper left panel of Fig. 3.^6^ For this estimate, we bin the 1000 points of the first test, and we calculate the standard deviation, $\sigma _{\chi ^2}$, for points with ${\chi ^2(\mathrm{LHC8}_\mathrm{col})}\le 1$, $1<{\chi ^2(\mathrm{LHC8}_\mathrm{col})}\le 4$ and ${\chi ^2(\mathrm{LHC8}_\mathrm{col})}>4$. We then apply the $\mathrm{LHC8}_\mathrm{col}$ constraint in three ways: with the nominal implementation, and shifting the ${\chi ^2(\mathrm{LHC8}_\mathrm{col})}$ penalty up and down according to these binned standard deviations. The results are shown in the upper left panel of Fig. 3 as solid and dotted red (blue) contours in the $(m_{\tilde{g}}, m_{\tilde{q}})$ plane corresponding to the nominal and up- and down-shifted cases for the 68 (95) % CL, respectively.^7^ A dedicated study of points within the 68 and 95 % CL regions confirms that our implementation of the $\mathrm{LHC8}_\mathrm{col}$ constraint is valid within these uncertainties, and our estimate of $\chi ^2$ at the best-fit point differs from the Scorpion evaluation by less than one.^8^

We conclude that the uncertainty $\sigma _{\chi ^2}$ in our estimate ${\chi ^2(\mathtt{Scorpion})}$ is generally reliable, and translates into an uncertainty of $\mathcal{O}(50\,\, \mathrm {GeV})$ in the limits on the gluino and squark masses, which is fully sufficient for the purpose of our studies.

#### LHC constraints on electroweak gauginos, Higgsinos and sleptons

Unlike the searches for coloured sparticles, where we were able to construct a computationally efficient, approximately universal limit, the LHC constraints on electroweakly produced sparticles vary strongly in sensitivity, depending on the mass hierarchy of sparticles and their corresponding decay modes and final states. For example, searches in the three-lepton plus missing energy channel constrain the chargino and neutralino masses up to $m_{\tilde{\chi }^\pm _{1}} = m_{\tilde{\chi }^0_{2}} \lesssim 700$ GeV for $m_{\tilde{\chi }^0_{1}} \lesssim 300$ GeV, if $\tilde{\chi }^\pm _{1}$ and $\tilde{\chi }^0_{2}$ decay exclusively into on-shell sleptons [116, 117], whereas a much weaker limit, $m_{\tilde{\chi }^\pm _{1}} = m_{\tilde{\chi }^0_{2}} \lesssim 450$ GeV for $m_{\tilde{\chi }^0_{1}} \lesssim 100$ GeV, was found in an analysis of the two-lepton plus missing energy channel [117, 118], assuming that the $\tilde{\chi }^\pm _{1}$ and $\tilde{\chi }^0_{2}$ decay exclusively into the $\tilde{\chi }^0_{1}$ in association with *W* and *Z*, respectively, and not taking into account the decay $\tilde{\chi }^0_{2} \rightarrow \tilde{\chi }^0_{1} h$ [119, 120]. The same two-lepton analyses constrain slepton pair production, leading to the limits $m_{\tilde{\ell }_{\mathrm{L(R)}}} \lesssim 270$ (200) GeV for $m_{\tilde{\chi }^0_{1}} \lesssim 100$ (50) GeV [117, 118]. Therefore, the universal limit approach that we use to combine and characterise searches for coloured sparticles is inapplicable to searches for electroweakly produced sparticles, and we use an alternative method.

For model points where the production of electroweakly produced sparticles provides a non-trivial constraint, they must be much lighter than the coloured sparticles, since otherwise the much higher rates of production of coloured sparticles would already exclude the model points. Therefore, in the region of interest, there can be only a few particles lighter than the electroweakly produced sparticles, implying that one can use a combination of a few simplified models (SMS) to approximate the sensitivities of the LHC searches for the production of these sparticles. Depending on the decay mode and final state, we select ATLAS and/or CMS limits derived from relevant simplified models to calculate the contributions of these searches to our global $\chi ^2$ function. For the LHC searches that constrain electroweakly produced gauginos, Higgsinos and sleptons, to a good approximation all relevant $\chi ^2$ contributions can be extracted from simplified chargino-neutralino and simplified smuon and selectron models.

For each simplified model limit we construct a function $\chi _\mathrm{SMS}^2$ that depends on the two relevant masses: ($m_{\tilde{\chi }^\pm _{1}}\simeq m_{\tilde{\chi }^0_{2}}, m_{\tilde{\chi }^0_{1}}$) for the simplified chargino–neutralino model and ($m_{\tilde{\ell }}, m_{\tilde{\chi }^0_{1}}$) for the simplified slepton $(\tilde{\ell }_{} \equiv \tilde{e}, \tilde{\mu })$ model. We assume that $\chi _\mathrm{SMS}^2 = 15$ in the bulk of the region excluded in the simplified model, and that this $\chi ^2$ penalty vanishes exponentially when crossing the boundary to the allowed region, with the general form

2 $$\begin{aligned} \chi _\mathrm{SMS}^2 \; = \; \min _{\mathrm{l,r}} \left[ 15\cdot B \cdot \frac{1}{\mathrm{e}^{(d_{\mathrm{l,r}}-\mu _{\mathrm{l,r}})/\sigma _{\mathrm{l,r}}}+1} \right] , \end{aligned}$$

where the subscripts l, r refer to points on the simplified model exclusion contour to the left (right) of the point with the largest value of $m_{\tilde{\chi }^0_{1}}$ [i.e., with smaller (larger) $m_{\tilde{\chi }^\pm _{1}}\simeq m_{\tilde{\chi }^0_{2}}$ or $m_{\tilde{\ell }}$], *B* is the branching ratio of the decay in question (as calculated with SDECAY [80]), *d* is the closest distance in $\,\, \mathrm {GeV}$ to the contour [with $d_{\mathrm{l,r}}$ positive to the left (right)], and $\mu$ and $\sigma$ control the precise fall-off of the $\chi ^2$ function, so as to mimic the experimental uncertainty bands, and they are functions of $m_{\tilde{\chi }^0_{1}}$. We note that if one sets $\mu =-\sigma$ then $\chi ^2_\mathrm{SMS}(d=0)\approx 4$, so that the exclusion on the contour corresponds approximately to the 95 % $\mathrm{CL}_s$. Finally, to avoid an unphysically slow fall-off outside the 95 % $\mathrm{CL}_s$ limit we set $\sigma =50\,\, \mathrm {GeV}$ and adjust *d* accordingly if $\sigma >50\,\, \mathrm {GeV}$ and $d-\mu >\sigma$ (and hence $\chi ^2_\mathrm{SMS}\lesssim 4$).

**Fig. 4:**
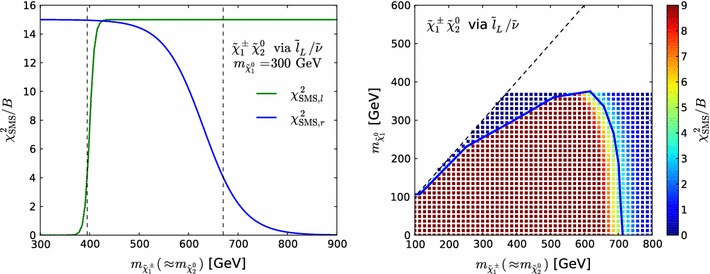
Illustration of $\chi ^2_\mathrm{SMS}{/}B$, as defined in Eq. (2), for $\tilde{\chi }^\pm _{1} \tilde{\chi }^0_{2}$ production and decay via sleptons. In the *left panel* $\chi ^2_\mathrm{SMS}/B$ is shown for a fixed value of $m_{\tilde{\chi }^0_{1}}=300\,\, \mathrm {GeV}$, where the *green (blue) line* corresponds to $d_\mathrm{l}, \mu _\mathrm{l},\sigma _\mathrm{l}$, ($d_\mathrm{r}, \mu _\mathrm{r}, \sigma _\mathrm{r}$) and *vertical dashed lines* indicate the position of the contour. The *right panel* shows the same $\chi ^2_\mathrm{SMS}/B$ (in *colour*) as a function of $m_{\tilde{\chi }^\pm _{1}}\simeq m_{\tilde{\chi }^0_{2}}$ and $m_{\tilde{\chi }^0_{1}}$, and the 95 % $\mathrm{CL}_s$ exclusion contour found in Fig. 7(a) of [116] (*blue line*)

In order to illustrate Eq. (2), we display in Fig. 4$\chi ^2_\mathrm{SMS}/B$ for the $\tilde{\chi }^\pm _{1} \tilde{\chi }^0_{2}$ decay via sleptons. In the left panel $\chi ^2_\mathrm{SMS}/B$ is shown for a fixed value of $m_{\tilde{\chi }^0_{1}}=300\,\, \mathrm {GeV}$ where the green (blue) line corresponds to $d_\mathrm{l}, \mu _\mathrm{l},\sigma _\mathrm{l}$, ($d_\mathrm{r}, \mu _\mathrm{r}, \sigma _\mathrm{r}$), whereas vertical dashed lines indicate the position of the contour. The right panel shows the same $\chi ^2_\mathrm{SMS}/B$ (in colour) as a function of $m_{\tilde{\chi }^\pm _{1}}\simeq m_{\tilde{\chi }^0_{2}}$ and $m_{\tilde{\chi }^0_{1}}$, and the 95 % $\mathrm{CL}_s$ exclusion contour found in Fig. 7(a) of [116] (blue line). Note that we apply no constraint for $m_{\tilde{\chi }^0_{1}} \gtrsim 380\,\, \mathrm {GeV}$, the highest value on the blue experimental contour.

In order to establish $\mathrm{LHC8}_\mathrm{EWK}$ we tuned the $\mu$ and $\sigma$ parameters for each simplified model to reproduce best the $\chi ^2$ values that we obtained using Atom for a representative set of model points from our sample. Table 2 summarises the implementations of the simplified model exclusion limits that contribute to $\mathrm{LHC8}_\mathrm{EWK}$. Note that, as described above, the large value of $\sigma _\mathrm{r}=300\,\, \mathrm {GeV}$ for the limit from $\tilde{\chi }^\pm _{1} \tilde{\chi }^0_{2}$ production and decay via *WZ* is replaced by setting $\sigma _\mathrm{r}=50\,\, \mathrm {GeV}$ and adjusting $d_\mathrm{r}$ accordingly when $d_\mathrm{r}-\mu _\mathrm{r}>\sigma _\mathrm{r}$ (and hence $\chi ^2_\mathrm{SMS}\lesssim 4$). Also, we had to produce our own contour for the direct production of right- and left-handed sleptons (selectrons and smuons), corresponding to their production cross sections. Note that this simplified model contour is also applied when left-handed sleptons decay via $\tilde{\chi }^0_{2}$ and $\tilde{\chi }^\pm _{1}$.

**Table 2:** The simplified model limits used to constrain electroweak gauginos, Higgsinos and sleptons

Simplified model	Limit	$(\mu _\mathrm{l}, \sigma _\mathrm{l})$ (GeV)	$(\mu _\mathrm{r}, \sigma _\mathrm{r})$ (GeV)
$\tilde{\chi }^\pm _{1} \tilde{\chi }^0_{2}$ via $\tilde{\ell }_{}$	Fig. 7(a) in [116]	$(-5, 5)$	$(-40, 40)$
$\tilde{\chi }^\pm _{1} \tilde{\chi }^0_{2}$ via *WZ*	Fig. 7(b) in [116]	$(-20, 20)$	$(-300, 300)$
$\tilde{\ell }_{} \rightarrow \ell \tilde{\chi }^0_{1,2},~\nu _\ell \tilde{\chi }^\pm _{1}$	Generated using Atom	$(-20, 10)$	$(-40, 30)$

**Fig. 5:**
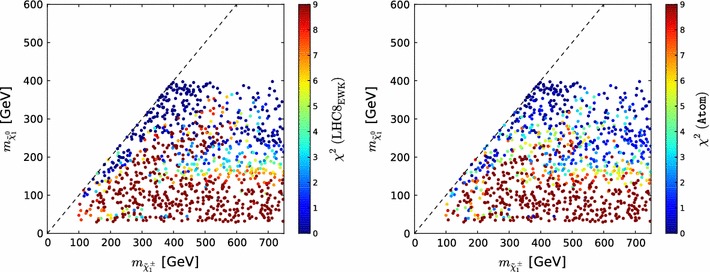
Scatter plots in the $(m_{\tilde{\chi }^\pm _{1}}, m_{\tilde{\chi }^0_{1}})$ plane of the contributions to the global $\chi ^2$ functions from the electroweakly interacting sparticle constraints for 1000 randomly selected points accessible to LHC searches, as calculated using the $\mathrm{LHC8}_\mathrm{EWK}$ method based on simplified model searches (${\chi ^2(\mathrm{LHC8}_\mathrm{EWK})}$, *left panel*) and the Atom code (${\chi ^2(\mathtt{Atom})}$, *right panel*)

In order to validate our method and to determine quantitatively its uncertainty, we compare the contributions to the global $\chi ^2$ function calculated with this $\mathrm{LHC8}_\mathrm{EWK}$ limit approach,

3 $$\begin{aligned} {\chi ^2(\mathrm{LHC8}_\mathrm{EWK})}&= \sum _\mathrm{SMS} \chi ^2_\mathrm{SMS}, \end{aligned}$$

to results from a full recast of all the above-listed searches as implemented in Atom. In this recast the full analysis is simulated, so that it is possible to determine for any arbitrary SUSY spectrum the $\mathrm{CL}_s$ value (and hence the corresponding $\chi ^2$) with which a given search penalises the SUSY spectrum. We obtain a set of 1000 model points from our sample by binning the $(m_{\tilde{\chi }^0_{2}}\approx m_{\tilde{\chi }^\pm _{1}}, m_{\tilde{\chi }^0_{1}})$ plane in $100\times 100$ bins, selecting one point randomly per bin, and then take a random subset of 1000 of these points. This procedure was employed to ensure a representative set of the decay modes in our sample.

Figure 5 displays scatter plots in the $(m_{\tilde{\chi }^\pm _{1}}, m_{\tilde{\chi }^0_{1}})$ plane of the contributions to the global $\chi ^2$ function for these 1000 model points as calculated using the $\mathrm{LHC8}_\mathrm{EWK}$ method (${\chi ^2(\mathrm{LHC8}_\mathrm{EWK})}$) (left panel) and the Atom code ${\chi ^2(\mathtt{Atom})}$ (right panel), with the indicated colour code in each plot. The immediate visual impression is that the colours in the two scatter plots are generally quite similar, indicating that the two procedures deliver similar $\chi ^2$ contributions overall. A closer inspection of the plots reveals similar bands of low-$\chi ^2$ points with small $m_{\tilde{\chi }^\pm _{1}} - m_{\tilde{\chi }^0_{1}}$ in a chargino coannihilation strip region, while elsewhere we see similar disfavouring of points with low $m_{\tilde{\chi }^0_{1}} \lesssim 150 \,\, \mathrm {GeV}$ and larger $m_{\tilde{\chi }^\pm _{1}}$. However, even within this band we see a sparse set of points with relatively low $\chi ^2$ that appear similarly in both the $\mathrm{LHC8}_\mathrm{EWK}$ analysis based on simplified models and the Atom implementation of the full searches. These are mainly due to the decay $\tilde{\chi }^0_{2} \rightarrow \tilde{\chi }^0_{1} h$, thus weakening the stronger $\tilde{\chi }^0_{2} \rightarrow \tilde{\chi }^0_{1} Z$-based limit.

**Fig. 6:**
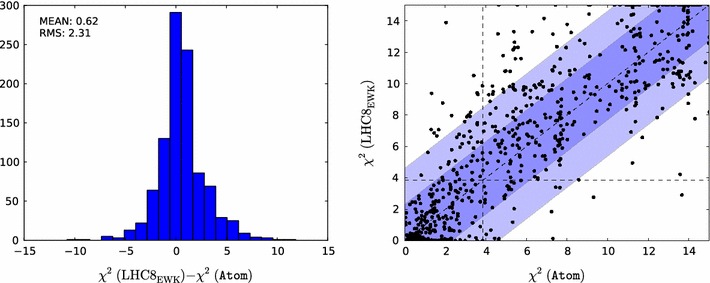
*Left panel* histogram of the differences between the values of the contributions of the electroweakly interacting sparticle constraints to the global likelihood function ${\chi ^2(\mathrm{LHC8}_\mathrm{EWK})}$ evaluated using simplified model searches for the 1000 randomly selected points and the estimate ${\chi ^2(\mathtt{Atom})}$ obtained using the Atom code. *Right panel* scatter plot in the $({\chi ^2(\mathtt{Atom})}, {\chi ^2(\mathrm{LHC8}_\mathrm{EWK})})$ plane of the $\chi ^2$ values obtained from the two approaches; the *vertical and horizontal dashed lines* in this plot correspond to the 95 % $\mathrm{CL}_s$ in each approach

For a more quantitative comparison of our $\mathrm{LHC8}_\mathrm{EWK}$ method and Atom we turn to Fig. 6. We see in the left panel that the difference between ${\chi ^2(\mathrm{LHC8}_\mathrm{EWK})}$ and ${\chi ^2(\mathtt{Atom})}$ is relatively small, with an r.m.s. difference $\sigma _{\chi ^2}=2.31$. The correlation between ${\chi ^2(\mathrm{LHC8}_\mathrm{EWK})}$ and $\chi ^2(\mathrm{Atom})$ is visible in the scatter plot in the right panel of Fig. 6. We see that most points are either excluded with $\Delta \chi ^2 > 4$ in both analyses, or allowed with $\Delta \chi ^2 < 4$ in both cases. Last but not least, there are relatively few ‘off-diagonal’ points with large $\Delta \chi ^2$, which form the small non-Gaussian tail of the ${\chi ^2(\mathrm{LHC8}_\mathrm{EWK})}$–${\chi ^2(\mathtt{Atom})}$ distribution seen in the left panel of Fig. 6.

To quantify the impact of this uncertainty on our analysis, we follow the same procedure as for our limits on coloured sparticles, and translate the $\sigma _{\chi ^2}$ (binned analogously) into a $\pm ~1~\sigma$ band for our 68 and 95 % CL contours in the important $(m_{\tilde{\chi }^\pm _{1}}, m_{\tilde{\chi }^0_{1}})$ and $(m_{\tilde{\mu }_{R}}, m_{\tilde{\chi }^0_{1}})$ planes. As can be seen in the upper right and lower left panels of Fig. 3, the uncertainty associated with $\mathrm{LHC8}_\mathrm{EWK}$ is in general small in the 68 % CL region of our fit, although it is larger at the 95 % CL level in the $(m_{\tilde{\chi }^\pm _{1}}, m_{\tilde{\chi }^0_{1}})$ plane. The effects on the best-fit point of these upward and downward shifts in the $\chi ^2$ treatment are shown in these panels as open green stars. The downward shift has very little effect, and is essentially invisible in the $(m_{\tilde{\chi }^\pm _{1}}, m_{\tilde{\chi }^0_{1}})$ plane. The upward shift increases the best-fit values of $m_{\tilde{\chi }^0_{1}}$ and $m_{\tilde{\chi }^\pm _{1}}$ while reducing that of $m_{\tilde{\mu }_{R}}$, though the variations are contained well within the 68 % CL region, clearly indicating that the corresponding uncertainties do not impact the overall conclusions.

#### LHC constraints on compressed stop spectra

In their searches for stop production, ATLAS and CMS have placed special emphasis on compressed spectra, which pose particular challenges for LHC searches. Whilst limits on stop production in the region where $m_{\tilde{t}_{1}} - m_{\tilde{\chi }^0_{1}} > m_t$ are fully included in the $\mathrm{LHC8}_\mathrm{col}$ limits described in Sect. 2.5.1, a dedicated treatment of the compressed-spectrum region $m_{\tilde{t}_{1}} - m_{\tilde{\chi }^0_{1}} < m_t$ is required in order to include properly all the relevant collider limits. In this region we calculate the contribution of stop searches to the global $\chi ^2$ in a similar way as for the for electroweakly produced sparticles described in Sect. 2.5.2. We refer to this dedicated limit-setting procedure as $\mathrm{LHC8}_\mathrm{stop}$.

**Fig. 7:**
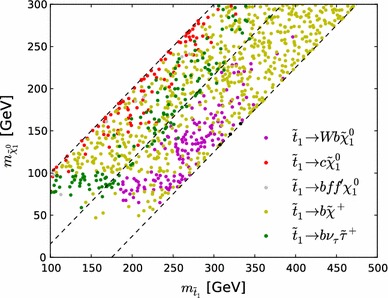
Scatter plot in the $(m_{\tilde{t}_{1}}, m_{\tilde{\chi }^0_{1}})$ plane of the $\tilde{t}_{1}$ decay modes with branching ratios $>$ $50$ % for 1000 randomly selected points with $m_{\tilde{t}_{1}} - m_{\tilde{\chi }^0_{1}} < m_t$

We show in Fig. 7 a colour-coded scatter plot in the $$(m_{\tilde{t}_{1}}, m_{\tilde{\chi }^0_{1}})$$ plane of the $$\tilde{t}_{1}$$ decay modes with branching ratios $$>$$$$50$$ % for 1000 randomly selected pMSSM10 points in the region of interest. We see that the $$\tilde{t}_{1} \rightarrow b \tilde{\chi }^\pm _{1}$$ mode (shown in light green) dominates for the majority of points, and that this decay can be important throughout the parameter region displayed. We also find that, when this is the dominant stop decay mode, in most cases the $$\tilde{\chi }^\pm _{1}$$ and $$\tilde{\chi }^0_{1}$$ are almost mass degenerate. To constrain the final states with this decay mode we implement the simplified model limit presented in Fig. 6 of the ATLAS di-bottom analysis [121], where $$m_{\tilde{\chi }^\pm _{1}} - m_{\tilde{\chi }^0_{1}} = 5$$ GeV is assumed, applying this for the model points with $$m_{\tilde{\chi }^\pm _{1}} - m_{\tilde{\chi }^0_{1}} < 30$$ GeV.

If $m_{\tilde{t}_{1}} - m_{\tilde{\chi }^0_{1}} > M_W+ m_b$, the 3-body $\tilde{t}_{1} \rightarrow b W \tilde{\chi }^0_{1}$ mode can dominate stop decay. The points for which this mode is dominant are shown by purple dots in Fig. 7. For this decay mode we implement the simplified model limit presented for $M_W+ m_b < m_{\tilde{t}_{1}} - m_{\tilde{\chi }^0_{1}} < m_t$ in Fig. 15 of the ATLAS single-lepton analysis [122].

**Table 3:** The simplified model limits used to constrain scenarios with compressed stop spectra. When establishing these limits we use values of $\mu _{\mathrm{l,r}}$ and $\sigma _{\mathrm{l,r}}$ in Eq. (2) that in some cases depend on $m_{\tilde{\chi }^0_{1}}$. Whenever multiple values of these parameters are specified for different values of $m_{\tilde{\chi }^0_{1}}$, the parameters for intermediate values of $m_{\tilde{\chi }^0_{1}}$ are obtained by linear interpolation, and they are taken as constants elsewhere

Decay	Limit	$m_{\tilde{\chi }^0_{1}}$ (GeV)	$(\mu _\mathrm{l}, \sigma _\mathrm{l})$ (GeV)	$(\mu _\mathrm{r}, \sigma _\mathrm{r})$ (GeV)	Condition/remark
$\tilde{t}_{} \rightarrow b \tilde{\chi }^\pm _{1}$	Fig. 6(c) in [121]	210	(10, 20)	$(-50, 50)$	$m_{\tilde{\chi }^\pm _{1}} - m_{\tilde{\chi }^0_{1}} < 30 \,\, \mathrm {GeV}$
		300	$(-250, 200)$	$(-200, 200)$	
$\tilde{t}_{} \rightarrow b W \tilde{\chi }^0_{1}$	Fig. 15 in [122]	100	$(-20,50)$	$(-70, 50)$	$M_W< m_{\tilde{t}_{1}} - m_{\tilde{\chi }^0_{1}} < m_t$
		150	$(-50, 50)$	$(-100,50)$	
$\tilde{t}_{} \rightarrow b \nu \tilde{\tau }_1$	Generated using Scorpion	–	$(-50, 50)$	$(-20, 50)$	Based on [110], assuming $m_{\tilde{\tau }_{1}} - m_{\tilde{\chi }^0_{1}} \lesssim 40 \,\, \mathrm {GeV}$
$\tilde{t}_{} \rightarrow c \tilde{\chi }^0_{1}$	Generated using Scorpion	–	$(-20, 20)$	$(-20, 20)$	Based on [110]

In the $m_{\tilde{t}_{1}} - m_{\tilde{\chi }^0_{1}} < M_W+ m_b$ region, the decays $\tilde{t}_{1} \rightarrow c \tilde{\chi }^0_{1}$ (red dots in Fig. 7) and $\tilde{t}_{1} \rightarrow b f f' \tilde{\chi }^0_{1}$ (grey dots) can be the dominant stop decay modes. The $\tilde{t}_{1} \rightarrow b \nu _\tau \tilde{\tau }_{1}$ mode (green dots) may also dominate stop decay in this region, as well as in the $m_{\tilde{t}_{1}} - m_{\tilde{\chi }^0_{1}} \gtrsim M_W+ m_b$ region, as can also be seen in Fig. 7.

Due to the variety of different stop decay modes that are relevant in this compressed region, we cannot use only the limits from simplified models provided by the experiments, as they do not cover all relevant decay chains and assume branching ratios of 100 %. However, these missing, in part rather complex, decay chains can effectively be constrained by hadronic inclusive searches such as those we have already used for our $\mathrm{LHC8}_\mathrm{col}$ limits. In particular, the CMS hadronic $m_{T2}$ search [110] has rather high sensitivity for these decay chains, as the kinematic phase space covered by the search makes no special assumptions on the final state, other than it having a purely hadronic signature.

Based on these inclusive searches, we derive limits for simplified models for $\tilde{t}_{1} \rightarrow c \tilde{\chi }^0_{1}$ and $\tilde{t}_{1} \rightarrow b \nu _\tau \tilde{\tau }_{1}$ decays. For the $\tilde{t}_{1} \rightarrow b \nu _\tau \tilde{\tau }_{1}$ simplified model we assume $m_{\tilde{\tau }_{1}} - m_{\tilde{\chi }^0_{1}} \lesssim 40$ GeV when creating the limit in the ($m_{\tilde{t}_{1}}$, $m_{\tilde{\chi }^0_{1}}$) plane. We do not implement a simplified model limit for $\tilde{t}_{1} \rightarrow b f f' \tilde{\chi }^0_{1}$ because this decay mode has negligible impact on our study, as can be seen in Fig. 7. Using these simplified model limits, we constrain the stop decay modes following a procedure very similar to what we used for $\mathrm{LHC8}_\mathrm{EWK}$, using an interpolating function of the form (2) to mimic the uncertainty (yellow) band in, e.g., Fig. 6c in [121]. We summarise our implementation of the simplified model limits in Table 3. When establishing these limits we use values of the parameters $\mu _{\mathrm{l,r}}$ and $\sigma _{\mathrm{l,r}}$ that depend on $m_{\tilde{\chi }^0_{1}}$. Whenever multiple values of these parameters are given for different values of $m_{\tilde{\chi }^0_{1}}$, the parameters for intermediate values of $m_{\tilde{\chi }^0_{1}}$ are obtained by linear interpolation, and they are taken as constants elsewhere.

**Fig. 8:**
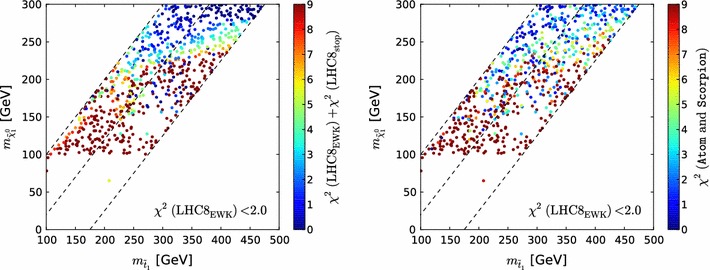
Scatter plots in the $(m_{\tilde{t}_{1}}, m_{\tilde{\chi }^0_{1}})$ plane of the contributions to the global $\chi ^2$ functions from the ATLAS monojet [123] and single-lepton [122] searches for 1000 randomly selected points in the regions of interest. The *left panel* shows calculations using simplified model searches (${\chi ^2(\mathrm{LHC8}_\mathrm{EWK})}$) and the *right panel* shows results from the Scorpion and Atom codes ($\chi ^2(\mathrm{true})$)

As for our $\mathrm{LHC8}_\mathrm{col}$ and $\mathrm{LHC8}_\mathrm{EWK}$ limit implementations, it is also important to determine accurately the uncertainty in the dedicated limit procedure for the compressed stop region. Note that in the compressed region not only the constraints from $\mathrm{LHC8}_\mathrm{stop}$ but also those from $\mathrm{LHC8}_\mathrm{EWK}$ play a role. Therefore we first assess the qualitative agreement between $\chi ^2(\mathrm{LHC8}_\mathrm{stop})$ and the “true” $\chi ^2(\mathtt{Atom}\mathrm{~and~}\mathtt{Scorpion})$ as calculated using the Scorpion and Atom codes, for points with ${\chi ^2(\mathrm{LHC8}_\mathrm{EWK})}<2$. Figure 8 compares scatter plots in the $(m_{\tilde{t}_{1}}, m_{\tilde{\chi }^0_{1}})$ plane of ${\chi ^2(\mathrm{LHC8}_\mathrm{EWK})}+{\chi ^2(\mathrm{LHC8}_\mathrm{stop})}$ (left panel) and $\chi ^2(\mathtt{Atom}\mathrm{~and~}\mathtt{Scorpion})$ (right panel). The colour code used is indicated on the right-hand sides of the panels, and we see that the patterns of colours in the two scatter plots are qualitatively similar. This is remarkable, given the interplay of so many different decay chains.

**Fig. 9:**
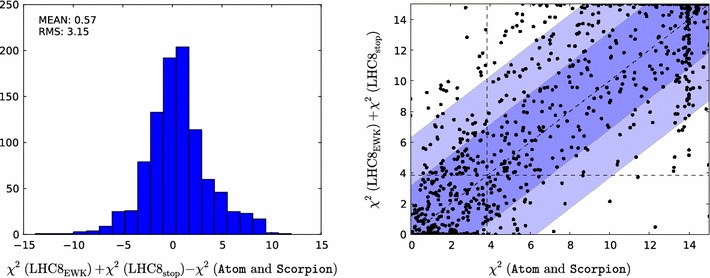
*Left panel* histogram of the difference between the values of the contributions of the stop constraints to the global likelihood function ${\chi ^2(\mathrm{LHC8}_\mathrm{EWK})}+{\chi ^2(\mathrm{LHC8}_\mathrm{stop})}$ evaluated using simplified model searches for 1000 randomly selected points and the estimates of $\chi ^2$ found using Scorpion and Atom. *Right panel* scatter plot in the $(\chi ^2(\mathrm{true}), {\chi ^2(\mathrm{LHC8}_\mathrm{EWK})}+{\chi ^2(\mathrm{LHC8}_\mathrm{stop})})$ plane of the values obtained from the two approaches; the *vertical and horizontal dashed lines* in these plots correspond to the 95 % $\mathrm{CL}_s$ in each approach

More quantitative comparisons of the contributions to the global $\chi ^2$ function calculated on the basis of the simplified model searches for stops and electroweakly produced sparticles (${\chi ^2(\mathrm{LHC8}_\mathrm{EWK})}+{\chi ^2(\mathrm{LHC8}_\mathrm{stop})}$) with results from Scorpion and Atom for these 1000 randomly selected pMSSM10 points ($\chi ^2(\mathrm{true})$) are shown in Fig. 9. The left panel shows a histogram of the difference between ${\chi ^2(\mathrm{LHC8}_\mathrm{EWK})}+{\chi ^2(\mathrm{LHC8}_\mathrm{stop})}$ and $\chi ^2(\mathrm{true})$, showing that it is relatively small, with an r.m.s. difference $\sigma _{\chi ^2}=3.15$. The right panel of Fig. 9 displays a scatter plot in the $({\chi ^2(\mathtt{Atom}\mathrm{~and~}\mathtt{Scorpion})}, {\chi ^2(\mathrm{LHC8}_\mathrm{EWK})}+{\chi ^2(\mathrm{LHC8}_\mathrm{stop})})$ plane. We see that points that are (dis)favoured at the 95 % $\mathrm{CL}_s$ level in the simplified approach are, in general, also (dis)favoured at the 95 % $\mathrm{CL}_s$ level in the more sophisticated approach based on Scorpion and Atom.

To determine quantitatively the effect of the uncertainty in the $\mathrm{LHC8}_\mathrm{stop}$ procedure, we translate the impact of the above-mentioned $\sigma _{\chi ^2}=3.15$ uncertainty into the $(m_{\tilde{t}_{1}}, m_{\tilde{\chi }^0_{1}})$ plane in the lower right panel of Fig. 3. This shows the impacts of $\pm$1 $\sigma _{\chi ^2}$ variations on our 68 and 95 % contours in this plane, which is rather small except for small values of $m_{\tilde{t}_{1}}$ and $m_{\tilde{\chi }^0_{1}}$.

Based on this study, we conclude that the computationally manageable simplified approach $\mathrm{LHC8}_\mathrm{stop}$ is sufficiently reliable for our physics purposes. Specifically, we note that there are points with low $m_{\tilde{t}_{1}}$ that survive the full LHC constraints with relatively low $\chi ^2$.

## Results

### Mass planes

**Fig. 10:**
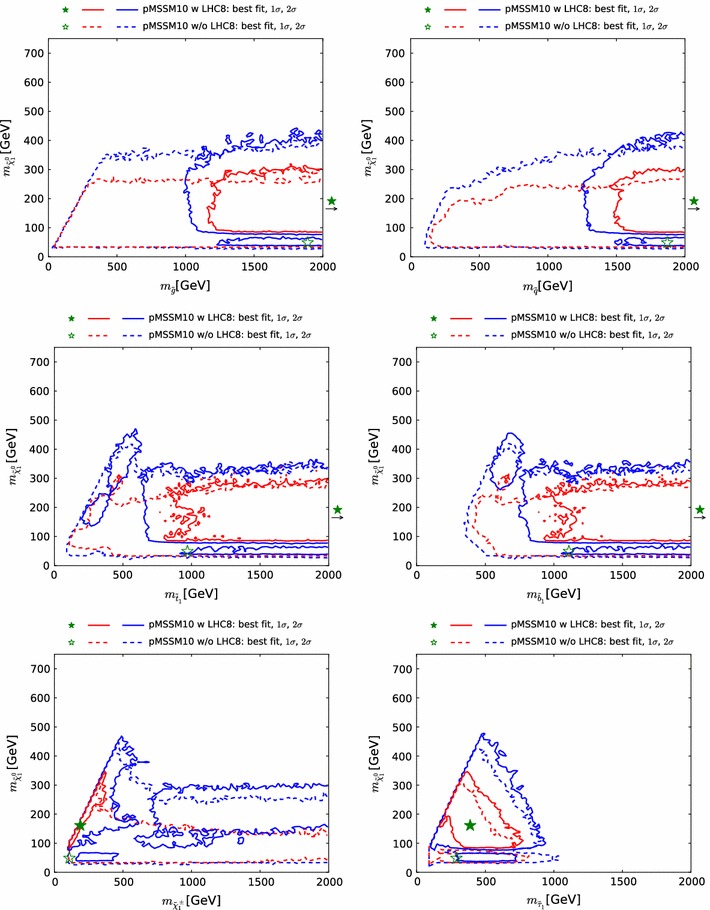
The two-dimensional profile likelihood functions for (*top left to bottom right*) the masses of the gluino, the first- and second-generation squarks, the lighter stop and sbottom squarks, the lighter chargino and the lighter stau, each versus the lightest neutralino mass $m_{\tilde{\chi }^0_{1}}$. In *each panel the solid* (*dashed*) *red/blue contours* denote the $\Delta \chi ^2 = 2.30/5.99$ level contours for the case where we do (not) apply the LHC8 constraints, respectively. The *green filled* and *empty stars* indicate the corresponding best-fit points

Figure 10 displays the two-dimensional profile likelihood functions in planes of (from top left to bottom right) the masses of the gluino, the first- and second-generation squarks, the lighter stop and sbottom squarks, the lighter chargino and the lighter stau, each versus the lightest neutralino mass $m_{\tilde{\chi }^0_{1}}$. In each panel the solid (dashed) red/blue contours denote the 68 %/95 % CL contours for the case where we do (not) apply any LHC constraints, respectively.^9^ The green filled and empty stars indicate the corresponding best-fit points. In the cases of the gluino and squarks, the filled stars lie beyond the displayed parts of the corresponding planes, and their locations are indicated by arrows. In these cases the likelihood function varies little as a function of the coloured sparticle mass.

On the other hand, we find that in general $$m_{\tilde{\chi }^0_{1}} \lesssim 300\,\, \mathrm {GeV}$$ at the $$\sim $$$$68~\%$$ CL, increasing to $$\sim 500 \,\, \mathrm {GeV}$$ at the $$\sim $$$$95~\%$$ CL. This and the preference for low stau masses ($$\lesssim $$$$700\,\, \mathrm {GeV}$$ at the $$\sim $$$$68~\%$$ CL, $$\lesssim $$$$1000 \,\, \mathrm {GeV}$$ at the $$\sim $$$$95~\%$$ CL) are reflections of the fulfilment of the $$(g-2)_\mu $$ constraint in the pMSSM10, cf., Fig. 15 below, and (in the latter case) the restriction to a common slepton mass for all three generations.

We can distinguish two ranges of $m_{\tilde{\chi }^0_{1}}$ that are allowed at the 95 % CL: a narrow band where $m_{\tilde{\chi }^0_{1}} \lesssim 80\,\, \mathrm {GeV}$ and a broader region at larger $m_{\tilde{\chi }^0_{1}}$ that also includes regions favoured at the 68 % CL. In the low-$m_{\tilde{\chi }^0_{1}}$ region, before applying the LHC8 constraints the smuon, selectron and stau could have been relatively light, and *t*-channel sfermion exchange could bring the relic density into the range allowed by cosmology. However, after applying the LHC8 constraints only the *Z*- and *h*-funnels are allowed in this region. In the region where $m_{\tilde{\chi }^0_{1}}\gtrsim 80\,\, \mathrm {GeV}$, before implementing the LHC8 constraints stau coannihilation and *t*-channel sfermion exchange were both possible. However, after applying the LHC8 constraints the dominant processes controlling the dark matter density are $\tilde{\chi }^0_{1} - \tilde{\chi }^0_{2} - \tilde{\chi }^\pm _{1}$ coannihilations, with the LSP having mainly a Bino composition.

The two top panels of Fig. 10 display clearly the direct impacts of the LHC8 constraints, which are visible in the displacements to larger masses of the $$68~\%$$ and $$95~\%$$ CL contours, as can be seen from the comparison of the solid and dashed lines. Our use of a comprehensive set of LHC searches including the CMS monojet and MT2 analyses as well as generic searches for  events gives us confidence that we model correctly the likelihood function also for gluino–$$\tilde{\chi }^0_{1}$$ and first- and second-generation squark–$$\tilde{\chi }^0_{1}$$ mass differences $$\lesssim $$$$40 \,\, \mathrm {GeV}$$, so that there are no unexcluded ‘islands’ with small values of these mass differences. On the other hand, the pictures in the two middle panels are more complex. There are intermediate values of $$m_{\tilde{t}_{1}}$$ that are disfavoured by the LHC8 constraints, but there are regions with low values of $$m_{\tilde{t}_{1}}$$ that are allowed by the LHC8 constraints at the 95 % CL, and even some points with $$m_{\tilde{t}_{1}}$$ and $$m_{\tilde{b}_{1}}$$ that are favoured at the 68 % CL, though these are not prominent. In the case of the lighter sbottom, the LHC8 constraints disfavour the region where both $$m_{\tilde{b}_{1}}$$ and $$m_{\tilde{\chi }^0_{1}}$$ have small values. However, a small value of $$m_{\tilde{b}_{1}}$$ is still allowed at the $$\sim $$$$95~\%$$ CL if $$m_{\tilde{\chi }^0_{1}} \gtrsim 300 \,\, \mathrm {GeV}$$ to $$450 \,\, \mathrm {GeV}$$, where some points are favoured at the 68 % CL.

Finally, the bottom two panels of Fig. 10 show the impacts of the LHC8 constraints on the chargino and stau masses. The main impact on the chargino mass is to disfavour most values except some where $$m_{\tilde{\chi }^\pm _{1}} - m_{\tilde{\chi }^0_{1}}$$ is small. This is an indirect effect of the LHC8 constraints, with the coannihilation of the dark matter particle with the lighter chargino playing an important role in bringing the dark matter density into the allowed range. This compression of the spectrum can be attributed to the $$\mathrm{LHC8}_\mathrm{EWK}$$ limits on direct production of light sleptons, and to a lesser extent on charginos decaying via sleptons. These constraints on light sleptons disfavour the *t*-channel sfermion exchange and stau coannihilation regions. The latter is a consequence of our choice of a single mass parameter for the masses of all the scalar leptons (see also Sect. 7). In the case of the lighter stau, we see in the bottom right panel of Fig.10 a triangular region that is favoured at the $$\sim $$$$68~\%$$ CL, which is somewhat reduced and shifted towards higher mass values by the LHC8 constraints.

### The best-fit point

**Table 4:** Parameters of the pMSSM10 best-fit point and other comparison benchmark points at low $m_{\tilde{t}_{1}}$, low $m_{\tilde{q}}$ and/or $m_{\tilde{g}}$

Parameter	Best-fit	Low $m_{\tilde{t}_{1}}$	Low $m_{\tilde{q}}$	Low $m_{\tilde{g}}$	Low all
$M_1$ (GeV)	170	300	210	190	$-120$
$M_2$ (GeV)	170	310	220	200	160
$M_3$ (GeV)	2600	1660	3730	$-1070$	1700
$m_{\tilde{q}}$ (GeV)	2880	3700	1530	2430	1790
$m_{\tilde{q}_3}$ (GeV)	4360	720	1840	3780	1300
$m_{\tilde{l}}$ (GeV)	440	390	430	410	740
$M_A$ (GeV)	2070	3540	2810	2990	1350
*A* (GeV)	790	1790	2510	3000	1863
$\mu$ (GeV)	550	1350	640	530	190
$\tan \beta$	37.6	37.3	40.8	33.9	35.4

**Fig. 11:**
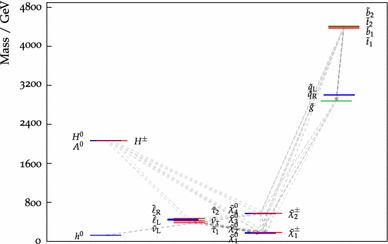
The particle spectrum and dominant decay branching ratios at our best-fit pMSSM10 point. Note the near-degeneracies between $\tilde{\chi }^0_{1}, \tilde{\chi }^0_{2}$ and $\tilde{\chi }^\pm _{1}$, between the sleptons, between $\tilde{\chi }^0_{3}, \tilde{\chi }^0_{4}$ and $\tilde{\chi }^\pm _{2}$, between the ${\tilde{q}_\mathrm{L}}$ and ${\tilde{q}_\mathrm{R}}$, between the heavy Higgs bosons, and between the stops and bottoms, which are general features of our 68 % CL region. On the other hand, the overall sparticle mass scales, in particular of the coloured sparticles, are poorly determined

**Fig. 12:**
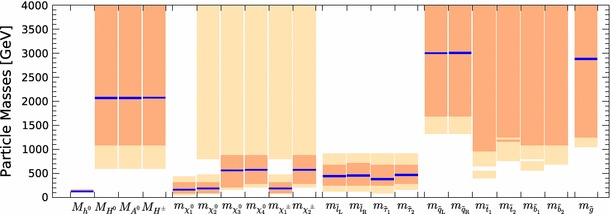
Summary of mass ranges predicted in the pMSSM10. The *light* (*darker*) *peach shaded bars* indicate the 95 % (68 %) CL intervals, whereas the *blue horizontal lines* mark the values of the masses at the best-fit point

**Table 5:** Table of the total $\chi ^2$ breakdowns at the pMSSM10 best-fit and low-$m_{\tilde{t}_{1}}$, low-$m_{\tilde{q}}$ and low-$m_{\tilde{g}}$ points, and in the CMSSM, NUHM1 and NUHM2 (updated from [53, 59], using in particular the current value of $M_h$ [94]). The $\mathrm{LHC8}_\mathrm{stop}$, $\mathrm{LHC8}_\mathrm{EWK}$ and $\mathrm{LHC8}_\mathrm{col}$ constraints were applied only to the pMSSM10, whereas a generic jets $+$  constraint was applied to the CMSSM, NUHM1 and NUHM2 [53, 59]. For each set of constraints, the (rounded) $\chi ^2$ contribution and the number of non-zero contributions is provided. The nuisance parameters are $m_t, \alpha _s(M_Z)$ and $M_Z$. The bottom rows show the number of parameters (including the nuisance parameters) and the total $\chi ^2/\mathrm{d.o.f.}$ omitting Higgs signal rates: the latter have been calculated only for the pMSSM10 points, and they are given separately in the last line. We also show an estimate of the corresponding $\chi ^2$ probability, which is calculated as the $\chi ^2$ probability neglecting correlations between the observables

Constraint	d.o.f.	pMSSM10					CMSSM	NUHM1	NUHM2
		best fit	low $m_{\tilde{t}_{1}}$	low $m_{\tilde{q}}$	low $m_{\tilde{g}}$	low all	[59]	[59]	[53]
LHC8	1	0.1	1.0	0.8	1.0	0.4	–	–	–
Jets$+$	1	–	–	–	–	–	2.0	0.0	0.5
$M_h$	1	0.0	0.0	0.2	0.2	0.0	0.1	0.1	0.4
$M_W$	1	0.0	0.1	0.1	0.5	0.1	0.0	0.0	0.4
$B_{s,d} \rightarrow \mu ^+ \mu ^-$	1	0.2	0.0	0.0	0.2	0.0	0.5	0.3	0.4
BR($b \rightarrow s \gamma$)	1	0.1	0.0	0.2	0.1	0.1	0.5	0.0	0.0
BR($B_u \rightarrow \tau \nu _\tau$)	1	0.2	0.2	0.2	0.2	0.3	0.2	0.2	0.2
Other *B* physics	5	3.3	3.0	3.2	3.3	3.0	3.2	3.3	3.3
$\Omega _{\tilde{\chi }^0_{1}} h^2$	1	0.1	0.0	0.3	0.0	0.1	0.0	0.0	0.0
$\sigma ^\mathrm{SI}_p$	1	0.0	0.1	0.0	0.1	0.5	0.0	0.0	0.0
$A/H\rightarrow \tau ^+\tau ^-$	1	0.0	0.0	0.0	0.0	0.0	0.0	0.0	0.0
Nuisance	3	0.0	0.1	0.0	0.1	0.8	0.1	0.0	0.1
$(g-2)_\mu$	1	0.0	0.7	0.0	0.0	0.6	9.3	10.6	8.4
*Z* pole	13	16.3	17.0	17.1	16.8	16.4	16.8	16.5	16.7
Parameters		10 $+$ 3	10 $+$ 3	10 $+$ 3	10 $+$ 3	10 $+$ 3	4 $+$ 3	5 $+$ 3	6 $+$ 3
$\chi ^2/\mathrm{d.o.f.}$		20.5/18	22.2/18	22.0/18	22.3/18	22.2/18	32.8/24	31.1/23	30.3/22
$\chi ^2$ probability		0.31	0.22	0.23	0.22	0.22	0.11	0.12	0.11
$\chi ^2(\mathrm{HS})$	77	62.8	62.6	62.8	62.8	62.8	–	–	–

We now discuss the characteristics of the best-fit point, whose parameters are listed in Table 4, together with the parameters of several benchmark points that are discussed below. The best-fit spectrum is shown in Fig. 11, and its SLHA file [86, 87] can be downloaded from the MasterCode website [63]. We note first the near-degeneracy between the $\tilde{\chi }^0_{1}, \tilde{\chi }^0_{2}$ and $\tilde{\chi }^\pm _{1}$, which is a general feature of our 68 % CL region that occurs in order to bring the cold dark matter density into the range allowed by cosmology: see the bottom left panel of Fig. 10. Correspondingly, we see in Table 4 that $M_1 \simeq M_2$, though $M_3$ is very different. The overall $\tilde{\chi }^0_{1}/\tilde{\chi }^0_{2}/\tilde{\chi }^\pm _{1}$ mass scale is bounded from below by the LEP and $\mathrm{LHC8}_\mathrm{EWK}$ constraints, and from above by $(g-2)_\mu$, especially at the 68 % CL. We display in Fig. 12 the 95 % (68 %) CL intervals in our fit for the masses of pMSSM10 particles as lighter (darker) peach shaded bars, with the best-fit values being indicated with blue horizontal lines.^10^ Turning back to Fig. 11, we note the near-degeneracy between the slepton masses, which reflects our assumption of a common input slepton mass at the input scale $M_\mathrm{SUSY}$, which would not hold in more general versions of the pMSSM. The overall slepton mass scale is below 1 $\,\, \mathrm {TeV}$, as seen in Fig. 12, being bounded from above by the $(g-2)_\mu$ and from below by the $\mathrm{LHC8}_\mathrm{EWK}$ constraint. The latter also provides the strongest upper bound on the $\tilde{\chi }^0_{1}/\tilde{\chi }^0_{2}/\tilde{\chi }^\pm _{1}$. We also see in Fig. 12 that the gluino, squark, stop and bottom masses are all very poorly constrained in our pMSSM10 analysis, though the $\mathrm{LHC8}_\mathrm{col}$ constraint forbids low masses.

Concerning the Higgs sector, we note that the best-fit value for $M_A$ lies in the multi-TeV region (where its actual value is only weakly constrained) and is therefore far in the decoupling region. Accordingly, the properties of the light Higgs boson at about 125 GeV resemble very closely those of the Higgs boson of the SM.

The first column of Table 5 lists the most important contributions to the total $\chi ^2$ function of different (groups of) constraints at the best-fit pMSSM10 point. The total $\chi ^2$ value at the best-fit point is $\chi ^2 = 83.3$, of which the largest part is due to the Higgs constraints evaluated using HiggsSignals.

To convert the total $\chi ^2$ of our fit into a $\chi ^2$ probability estimate, we calculate the $\chi ^2$ contribution and corresponding number of degrees of freedom (d.o.f.) by considering only constraints that have significant contributions to our global $\chi ^2$ function in large regions of the relevant parameter space. We do not include in this procedure constraints from HiggsSignals, which do not in general vary strongly in our preferred fit regions (see, e.g., $\chi ^2(HS)$ in Table 5). Therefore, to calculate the $\chi ^2$ probability we consider in total 31 constraints, which translate into 18 d.o.f for the pMSSM10, 24 d.o.f. for CMSSM, 23 d.o.f. for NUHM1 and 22 d.o.f. for NUHM2. Previous studies [125] showed that this definition of the $\chi ^2$ probability represents a good estimate of fit quality and enables a comparison between different models on an equal footing. It also represents a reasonable approximation to the underlying absolute *p* values of our fits.

Comparing to the $\chi ^2$ values for the CMSSM, NUHM1 and NUHM2 shown in the last three columns of Table 5, we see that the largest improvement is in the contribution from $(g-2)_\mu$, though there are also small improvements in $\mathrm{BR}(B_s \rightarrow \mu ^+\mu ^-)$ and the *Z*-pole observables. Overall, we see that the pMSSM10 has a $\chi ^2$ probability of 30.8 % compared to 10.8, 12.1 and 11.0 % for the CMSSM, NUHM1 and NUHM2, respectively, demonstrating that the pMSSM10 gives a significantly better fit.^11^

We stress, however, that these $\chi ^2$ probabilities are only approximate and assume an underlying $\chi ^2$-distribution with no correlations between the observables. A more proper treatment would be to smear the measurements around the best-fit predictions, fit to these toy measurements and evaluate the fraction of cases in which the resulting $\chi ^2$ exceeds the observed $\chi ^2$. We leave such an evaluation as a topic for future work.

### Sparticle masses

**Fig. 13:**
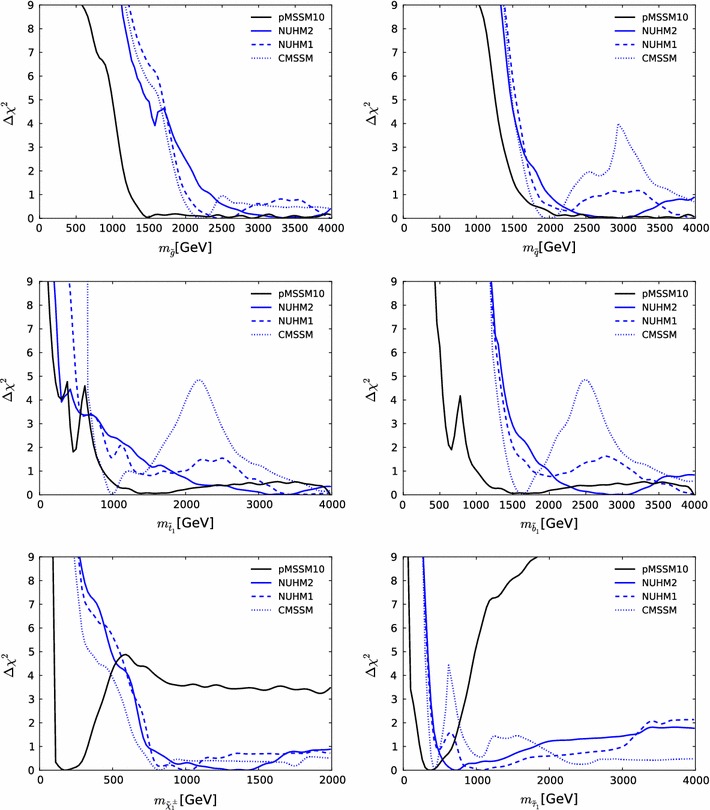
The one-dimensional profile likelihood functions for $m_{\tilde{g}}$, $m_{\tilde{q}}$, $m_{\tilde{t}_{1}}$, $m_{\tilde{b}_{1}}$, $m_{\tilde{\chi }^\pm _{1}}$ and $m_{\tilde{\tau }_{1}}$. In *each panel* the *solid black line* is for the pMSSM10, the *solid blue line* for the NUHM2, the *dashed blue line* for the NUHM1 and the *dotted blue line* for the CMSSM

Figure 13 displays (from top left to bottom right) the one-dimensional profile likelihood functions for the masses of the gluino, the first- and second-generation squarks, the lighter stop and sbottom squarks, the lighter chargino and the lighter stau. In each panel the solid black line is for the pMSSM10, the solid blue line for the NUHM2, the dashed blue line for the NUHM1 and the dotted blue line for the CMSSM (the latter three lines are updated from Ref. [53] to include new constraints such as the LHC combined value of $$M_h$$ [94]). In the case of $$m_{\tilde{g}}$$, we see that significantly lower masses are allowed in the pMSSM10 than in the other models: $$> 1250 \,\, \mathrm {GeV}$$ at the 68 % CL and $$\sim $$$$1000 \,\, \mathrm {GeV}$$ at the 95 % CL. We also see that there is a similar, though smaller, reduction in the lower limit on $$m_{\tilde{q}}$$, to $$\sim $$$$1500\,\, \mathrm {GeV}$$ at the 68 % CL and $$\sim $$$$1300\,\, \mathrm {GeV}$$ at the 95 % CL. The picture is more complicated for $$m_{\tilde{t}_{1}}$$, where we see structures in the one-dimensional likelihood function for $$m_{\tilde{t}_{1}} < 1000 \,\, \mathrm {GeV}$$ that reflect the low-mass islands in the corresponding panel of Fig. 10 that are allowed at the 95 % CL. In the bottom row of Fig. 13, the one-dimensional profile likelihood functions for $$m_{\tilde{\chi }^\pm _{1}}$$ and $$m_{\tilde{\tau }_{1}}$$ in the pMSSM have minima at the lower mass limits $$\sim $$$$100~\,\, \mathrm {GeV}$$ established at LEP, and there is an upper limit $$m_{\tilde{\tau }_{1}} \lesssim 1000 \,\, \mathrm {GeV}$$ at the 95 % CL. These effects are due to the $$(g-2)_\mu $$ constraint and the choice of generation-independent slepton masses in the pMSSM10. On the other hand, the light chargino (which is nearly degenerate in mass with the second lightest neutralino) has an upper mass limit below $$500 \,\, \mathrm {GeV}$$ at the 90 %, which would allow neutralino and chargino pair production at an 1000 GeV $$e^+e^-$$ collider, as we discuss later. However, we find no upper limit on $$m_{\tilde{\chi }^\pm _{1}}$$ at the 95 % CL.

### Benchmark pMSSM10 models

In view of the variety of pMSSM10 parameters that are allowed at the 68 % CL, we consider in this subsection various specific benchmark models that illustrate the range of possibilities. Specifically, looking at the middle panels of Fig. 10, we see that a very low stop mass in the compressed stop region is possible, and the top panels of Fig. 10 show the possibilities for a gluino *or* squark mass that is lower than at the best-fit point. Also, we see in the upper left panel of Fig. 3 that SUSY may well appear with *both* the squark and gluino masses having lower masses than at the best-fit point. We investigate these possibilities with the benchmark points discussed below, whose SLHA files [86, 87] can be downloaded from the MasterCode website [63].

#### Low-$m_{\tilde{t}_{1}}$ point

**Fig. 14:**
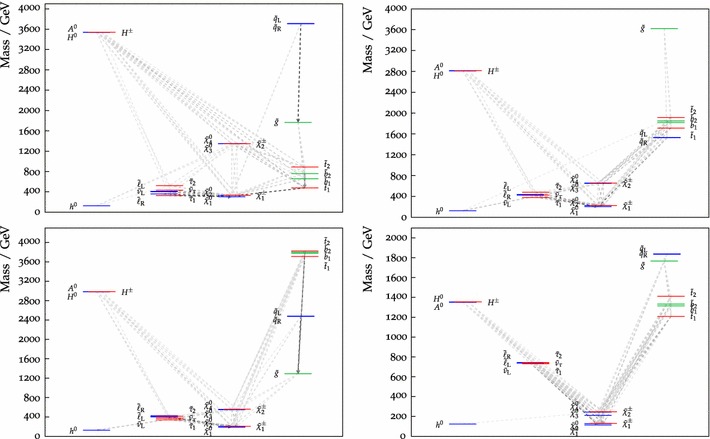
The particle spectra and dominant decay branching ratios at the benchmark points discussed in the text. *Upper left panel* the low-$m_{\tilde{t}_{1}}$ pMSSM10 point, where the stops and bottoms are relatively light. *Upper right panel* similarly for the low-$m_{\tilde{q}}$ benchmark point, where all the squarks are relatively light. *Lower left panel* similarly for the low-$m_{\tilde{g}}$ benchmark point. *Lower right panel* similarly for the point where all squarks and the gluino masses are $< 2 \,\, \mathrm {TeV}$. Note in each case the near-degeneracies between $\tilde{\chi }^0_{1}, \tilde{\chi }^0_{2}$ and $\tilde{\chi }^\pm _{1}$, between the sleptons, between $\tilde{\chi }^0_{3}, \tilde{\chi }^0_{4}$ and $\tilde{\chi }^\pm _{2}$, between the ${\tilde{q}_\mathrm{L}}$ and ${\tilde{q}_\mathrm{R}}$, and between the heavy Higgs bosons

We display in the upper left panel of Fig. 14 the spectrum at the point that minimises $\chi ^2$ locally within the low-$m_{\tilde{t}_{1}}$ (and low-$m_{\tilde{b}_{1}}$) 68 % CL region visible in the middle planes of Fig. 10. Like the pMSSM10 best-fit point shown in Fig. 11, this point also exhibits near-degeneracies between $\tilde{\chi }^0_{1}, \tilde{\chi }^0_{2}$ and $\tilde{\chi }^\pm _{1}$, between the sleptons, between $\tilde{\chi }^0_{3}, \tilde{\chi }^0_{4}$ and $\tilde{\chi }^\pm _{2}$ (reflected also in the fact that $M_1 \simeq M_2$, as seen in the second column of Table 4), and between the ${\tilde{q}_\mathrm{L}}$ and ${\tilde{q}_\mathrm{R}}$. However, all the stops and sbottoms are light at this point. As in Fig. 11, the dominant decay modes are illustrated in 50 shades of grey [126]. The second column of Table 5 lists the contributions to the total $\chi ^2$ function of different (groups of) constraints at this low-$m_{\tilde{t}_{1}}$ pMSSM10 point. Comparing with the corresponding breakdown for the best-fit point shown in the first column of Table 5, we see larger contributions from the LHC8 constraint (principally from $\mathrm{LHC8}_\mathrm{col}$) and from $(g-2)_\mu$, which are largely responsible for the increase in the total $\chi ^2$ to 22.2 (omitting the HiggsSignals contributions) and the corresponding decrease in the $\chi ^2$ probability to 0.22. However, we emphasise that this point provides a perfectly acceptable fit to all the constraints.

#### Low-$m_{\tilde{q}}$ point

We consider next a benchmark point with relatively low masses for the first- and second-generation squarks. As can be seen in the top right panel of Fig. 10, the lowest value of $m_{\tilde{q}}$ that is allowed at the 68 % CL is $\simeq 1500 \,\, \mathrm {GeV}$, and we have chosen as benchmark a point that also has $m_{\tilde{\chi }^0_{1}} \simeq 200 \,\, \mathrm {GeV}$, whose spectrum is shown in the upper right panel of Fig. 14. We see there that the near-degeneracies between $\tilde{\chi }^0_{1}, \tilde{\chi }^0_{2}$ and $\tilde{\chi }^\pm _{1}$, between the sleptons, between $\tilde{\chi }^0_{3}, \tilde{\chi }^0_{4}$ and $\tilde{\chi }^\pm _{2}$, and between the heavy Higgs bosons are very similar to those at the best-fit and low-$m_{\tilde{t}_{1}}$ points. By choice, the masses of the first- and second-generation squarks are much lighter than at either of these points, and the third-generation squarks have masses intermediate between the best-fit and low-$m_{\tilde{t}_{1}}$ points. As seen in Table 5, the largest part of the increase in $\chi ^2$ to 22.0, compared to the best-fit point, and the corresponding decrease in the $\chi ^2$ probability to 0.22, is again due to the LHC8 constraint.

#### Low-$m_{\tilde{g}}$ point

We consider next a benchmark point with a relatively low gluino mass. As can be seen in the top left panel of Fig. 10, our global fit requires $m_{\tilde{g}}\gtrsim 1250 \,\, \mathrm {GeV}$ at the 68 % CL. We have chosen as benchmark a point that has this value of $m_{\tilde{g}}$ and also $m_{\tilde{\chi }^0_{1}} \simeq 200 \,\, \mathrm {GeV}$, whose spectrum is shown in the right panel of Fig. 14. We see again the near-degeneracies within groups of MSSM particles, as for the benchmark points considered previously. We see a clear hierarchy of masses between the groups of strongly interacting sparticles, with the third-generation sparticles being much heavier than those of the first and second generation, which are in turn much heavier than the gluino. Again as seen in Table 5, the largest part of the increase in $\chi ^2 \rightarrow 22.3$ compared to the best-fit point is again due to the $\mathrm{LHC8}_\mathrm{col}$ constraint, with increases also from $\mathrm{LHC8}_\mathrm{EWK}$, and $M_W$. The total $\chi ^2$ probability of 21.7 % is comparable to those of the low-$m_{\tilde{t}_{1}}$ and -$m_{\tilde{q}}$ points.

**Fig. 15:**
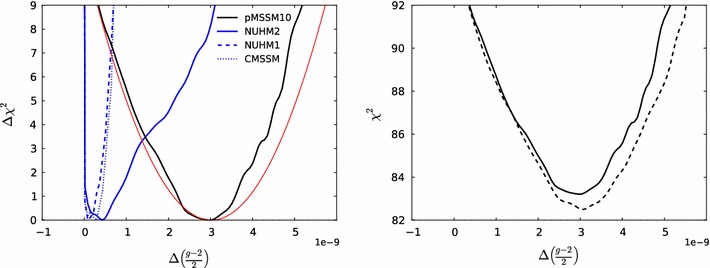
Profile likelihoods for the SUSY contribution to $(g-2)_\mu$. The *left panel* shows the $\Delta \chi ^2$ contributions from $(g-2)_\mu$ to the global likelihood functions of our fits to the CMSSM (*blue dotted line*), the NUHM1 (*blue dashed line*), the NUHM2 (*blue solid line*) and the pMSSM10 (*black solid line*), as well as the experimental likelihood function that we assume (*solid red line*). The *right panel* displays the global $\chi ^2$ function calculated without (*dashed line*) and with (*solid line*) the contribution of the electroweakly interacting sparticle searches implemented via $\mathrm{LHC8}_\mathrm{EWK}$

#### Point with squark and gluino masses below $2 \,\, \mathrm {TeV}$

Finally, we display in the lower right panel of Fig. 14 the spectrum at a point from near the turning-point in Fig. 3, which can be regarded as a ‘compromise’ between the two previous benchmarks where the gluino and all the squarks (including those in the third generation) have masses $< 2\,\, \mathrm {TeV}$, as do the heavy *A* / *H* Higgs bosons. Like the previous pMSSM10 benchmark points, this point also exhibits near-degeneracies between $\tilde{\chi }^0_{1}, \tilde{\chi }^0_{2}$ and $\tilde{\chi }^\pm _{1}$, between the sleptons, between $\tilde{\chi }^0_{3}, \tilde{\chi }^0_{4}$ and $\tilde{\chi }^\pm _{2}$ , and between the ${\tilde{q}_\mathrm{L}}$ and ${\tilde{q}_\mathrm{R}}$. In addition, the sbottom squarks are also nearly degenerate, whereas the stops exhibit a greater mass splitting, due to the $m_t$-dependence in the off-diagonal stop mass matrix elements. The contributions to the total $\chi ^2$ function of different (groups of) constraints at this low-mass pMSSM10 point are shown in the fifth column of Table 5. Comparing with the corresponding breakdown for the best-fit point, we see larger contributions from $\mathrm{LHC8}_\mathrm{col}$, $\sigma ^\mathrm{SI}_p$ and $(g-2)_\mu$. All these contributions to $\chi ^2$ are $<$1, but they do suggest possibilities for SUSY discovery in jets $+$ searches early during LHC run 2, and in upcoming direct dark matter detection experiments. The total $\chi ^2 = 22.2$ at this point, and the corresponding $\chi ^2$ probability is 22.4 %.

### The anomalous magnetic dipole moment of the muon $(g-2)_\mu$

It is well known that there is a discrepancy of $$\sim $$$$3.5\sigma $$ between the measured value of $$(g-2)_\mu $$ [51, 52] and the value predicted in the SM [127–136]. Sizeable contributions to $$(g-2)_\mu $$ from SUSY can occur when smuons, charginos and the lightest neutralino have masses of $$\mathcal{O}(100 \,\, \mathrm {GeV})$$. It is known from previous analyses of the CMSSM, NUHM1 and NUHM2 [53, 59] that in these models there is tension between SUSY interpretations of the discrepancy in the anomalous magnetic dipole moment of the muon $$(g-2)_\mu $$ (which favour lower electroweak sparticle masses) and LHC constraints from direct searches for sparticles and the measured value of the lightest Higgs boson (which favour higher coloured sparticle masses). This tension arises from the universality relations imposed in these models at the GUT scale between the soft SUSY-breaking contributions to the masses of the strongly and electroweakly interacting sparticles. In the pMSSM10 there are no such assumptions, and thus one might hope to resolve this tension.

This point is apparent in Fig. 15, where we display in the left panel the contributions $\Delta \chi ^2$ from $(g-2)_\mu$ to the global $\chi ^2$ functions of our fits to the CMSSM (blue dotted line), the NUHM1 (blue dashed line), the NUHM2 (blue solid line) and the pMSSM10 (black solid line), as well as the experimental likelihood function that we assume (solid red line). We see that the pMSSM10 is able to fit $(g-2)_\mu$ perfectly with $\Delta \chi ^2 \simeq 0$ at the best-fit point, whereas the other “universal” models exhibit contributions $\Delta \chi ^2 \gtrsim 9$ from $(g-2)_\mu$.

**Fig. 16:**
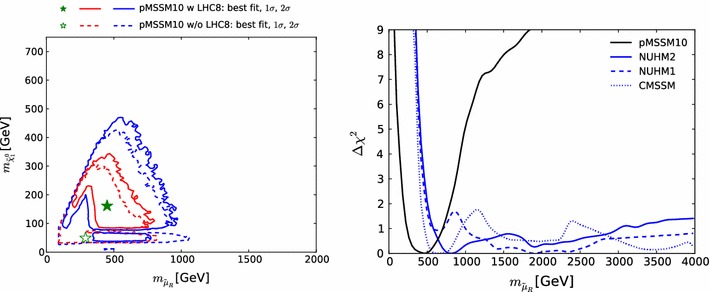
*Left panel* the two-dimensional profile likelihood function for $m_{\tilde{\mu }_{R}}$ versus the lightest neutralino mass $m_{\tilde{\chi }^0_{1}}$. The *solid* (*dashed*) *red/blue* contours denote the $\Delta \chi ^2 = 2.30/5.99$ level contours for the case where we do (not) apply the LHC8 constraints, respectively, and the *green filled* and *empty stars* indicate the corresponding best-fit points. *Right panel* the one-dimensional profile likelihood function for $m_{\tilde{\mu }_{R}}$ in the pMSSM10 (*solid black line*), the NUHM2 (*solid blue line*), the NUHM1 (*dashed blue line*) and the CMSSM (*dotted blue line*)

We display in the right panel of Fig. 15 the impact on the global $\chi ^2$ as a function of $(g-2)_\mu$ of implementing the LHC constraints on electroweakly interacting sparticles using the $\mathrm{LHC8}_\mathrm{EWK}$ method described earlier (which, as we have shown, provides a reasonably accurate as well as computationally economical representation of the LHC8 constraints on electroweakly interacting sparticles). The solid line is the global $\chi ^2$ function with the $\mathrm{LHC8}_\mathrm{EWK}$ constraint included, and the dashed line when they are omitted. The minimum value of $\chi ^2$ increases from 82.6 to 83.3, and the value of $(g-2)_\mu$ at the minimum is essentially unchanged. We conclude that the impacts of the LHC searches for electroweakly interacting particles are limited, and the pMSSM10 resolution of the $(g-2)_\mu$ puzzle survives the LHC electroweak constraints with flying colours.

The left panel of Fig. 16 displays the two-dimensional profile likelihood function in the $(m_{\tilde{\mu }_{R}}, m_{\tilde{\chi }^0_{1}})$ plane, with the solid (dashed) red/blue contours denoting the $\Delta \chi ^2 = 2.30/5.99$ level contours for the case where we do (not) apply the LHC8 constraints, respectively, and the green filled and empty stars indicating the corresponding best-fit points.^12^ Qualitatively, this plane is quite similar to the corresponding $(m_{\tilde{\tau }_1}, m_{\tilde{\chi }^0_{1}})$ plane shown in the bottom right panel of Fig. 10, though we note, e.g., that the best-fit value of $m_{\tilde{\mu }_{\mathrm{R}}}$ is $\sim 100 \,\, \mathrm {GeV}$ larger than the best-fit value of $m_{\tilde{\tau }_1}$. This feature is apparent also when one compares the right panel of Fig. 16, which displays the one-dimensional profile likelihood function for $m_{\tilde{\mu }_{\mathrm{R}}}$ with the corresponding plot for $m_{\tilde{\tau }_1}$ in the bottom right panel of Fig. 13. In both cases, the one-dimensional profile likelihood function in the pMSSM10 is shown as a solid black line, that in the NUHM2 as a solid blue line, that in the NUHM1 as a dashed blue line and that in the CMSSM as a dotted blue line.

### Interplay of the $\mathrm{LHC8}_\mathrm{EWK}$, $(g-2)_\mu$ and dark matter constraints

**Fig. 17:**
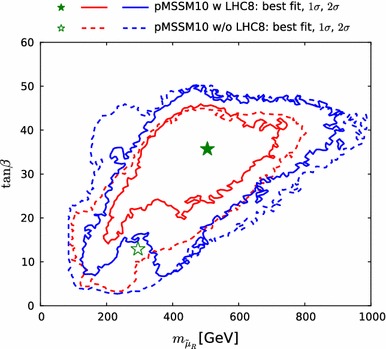
The 68 and 95 % CL regions in the $(m_{\tilde{\mu }_\mathrm{R}}, \tan \beta )$ plane before (*dashed lines*) and after (*solid lines*) implementation of the LHC8 and other constraints

The 68 and 95 % CL regions in the $(m_{\tilde{\mu }_\mathrm{R}}, \tan \beta )$ plane before (dashed lines) and after (solid lines) implementation of the LHC8 and other constraints are displayed in Fig. 17. We see that the lowest values of $\tan \beta$ receive a $\chi ^2$ penalty, which is due to a combination of different effects. In particular, the $\mathrm{LHC8}_\mathrm{EWK}$ constraint disfavours lower values of $m_{\tilde{\mu }_{\mathrm{R,L}}}$ which, in combination with $(g-2)_\mu$, results in a $\chi ^2$ penalty for $\tan \beta \lesssim 10$. Because we impose slepton mass universality in the pMSSM10, stau masses are also pushed to higher values. In this way the $\mathrm{LHC8}_\mathrm{EWK}$ constraints eliminate pMSSM10 models with a Bino-like LSP and small $\sigma ^\mathrm{SI}_p$, for which stau coannihilation and *t*-channel slepton exchanges brought the relic LSP density into the allowed range. The remaining models with $\tan \beta \lesssim 30$ then fall foul of the LUX upper limit [92] on $\sigma ^\mathrm{SI}_p$, because the LSP has a substantial Higgsino component, which enhances $\sigma ^\mathrm{SI}_p$. The overall combined effect of the $\mathrm{LHC8}_\mathrm{EWK}$, $(g-2)_\mu$ and dark matter constraints is to prefer values of $\tan \beta$ between about 15 and 45 at the 68 % CL, though $\tan \beta$ values below 10 are still allowed at the 95 % CL. We note that this feature is an effect of the choice of a single slepton mass scale, which could be avoided in more general versions of the pMSSM.

### Higgs physics

**Fig. 18:**
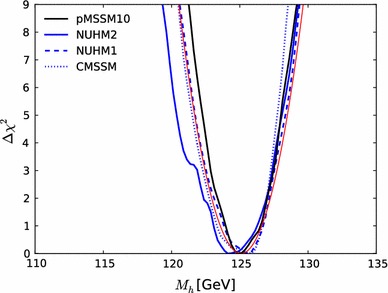
The one-dimensional profile likelihood function for $M_h$: the *solid black line* is for the pMSSM10, the *solid blue line* for the NUHM2, the *dashed blue line* for the NUHM1, the *dotted blue line* for the CMSSM and the *red line* is the $\chi ^2$ penalty from the experimental measurement of $M_h$ *with the assumed theoretical uncertainty of* $1.5 \,\, \mathrm {GeV}$

Figure 18 displays one-dimensional profile likelihood for $M_h$ when the LHC constraints are applied. We see that the likelihood for $M_h$ in the pMSSM10 (black line) is very similar to the experimental value smeared by the theoretical uncertainty in the FeynHiggs calculation of $M_h$ for specific values of the MSSM input parameters.

**Fig. 19:**
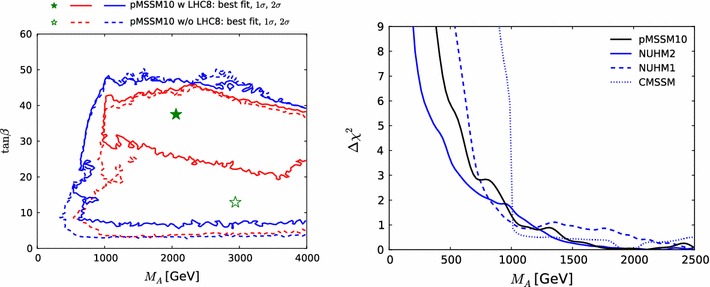
*Left panel* the two-dimensional profile likelihood function for $M_A$ versus $\tan \beta$. The *solid* (*dashed*) *red/blue* contours denote the $\Delta \chi ^2 = 2.30/5.99$ level contours for the case where we do (not) apply the LHC constraints, respectively, and the *green filled* and *empty stars* indicate the corresponding best-fit points. *Right panel* the one-dimensional profile likelihood function for $M_A$: the *solid black line* is for the pMSSM10, the *solid blue line* for the NUHM2, the *dashed blue line* for the NUHM1 and the *dotted blue line* for the CMSSM

The left panel of Fig. 19 displays the two-dimensional profile likelihood function in the $(M_A, \tan \beta )$ plane. As before, the solid (dashed) red/blue contours denote the $\Delta \chi ^2 = 2.30/5.99$ level contours for the case where we do (not) apply the LHC8 constraints, respectively, and the green filled and empty stars indicate the corresponding best-fit points. Comparing the dashed and solid 68 % contours, we see that lower values of $\tan \beta$ are disfavoured at the 68 % CL by the combination of $\mathrm{LHC8}_\mathrm{EWK}$, $(g-2)_\mu$ and Dark Matter constraints, as discussed in the previous subsection. Those constraints, in combination with the choice of a single slepton mass scale for all three generations, lead to limits of $M_A\gtrsim 1000 (500) \,\, \mathrm {GeV}$ at the 68 (95) % CL, whereas otherwise low CP-odd Higgs boson masses down to $M_A\sim 500 (350) \,\, \mathrm {GeV}$ would be found in the 68 (95) % CL area.

The right panel of Fig. 19 displays the corresponding one-dimensional profile likelihood function for $M_A$: as before, the solid black line is for the pMSSM10, the solid blue line for the NUHM2, the dashed blue line for the NUHM1 and the dotted blue line for the CMSSM. Lower $M_A$ values for $\tan \beta \lesssim 30$ are in particular disfavoured by the LUX and other limits, as discussed in the previous subsection.

### $\mathrm{BR}(B_s \rightarrow \mu ^+\mu ^-)$ decay

**Fig. 20:**
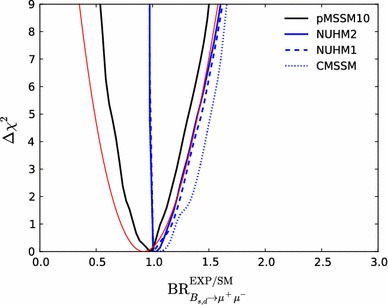
The one-dimensional profile likelihood functions for $\mathrm{BR}(B_s \rightarrow \mu ^+\mu ^-)$ relative to the SM value. The *solid black line* is for the pMSSM10, the *solid blue line* for the NUHM2, the *dashed blue line* for the NUHM1, the *dotted blue line* for the CMSSM, and the *red line* is the $\chi ^2$ penalty from the experimental constraint

**Fig. 21:**
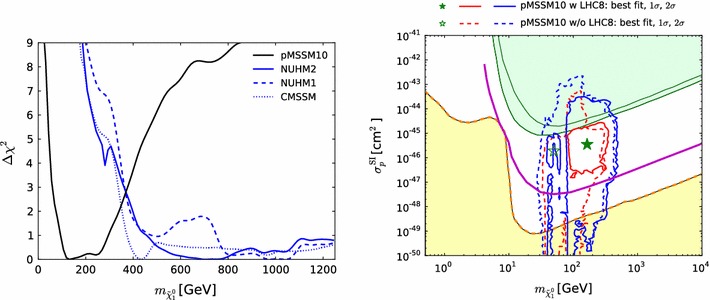
*Left panel* the one-dimensional profile likelihood in the pMSSM10 for $m_{\tilde{\chi }^0_{1}}$ (*black line*), compared with the NUHM2, the NUHM1 and the CMSSM (*solid*, *dashed* and *dotted blue lines*, respectively). *Right panel* the two-dimensional profile likelihood function in the pMSSM in the $(m_{\tilde{\chi }^0_{1}}, \sigma ^\mathrm{SI}_p)$-plane, showing the regions excluded by the XENON100 and LUX experiments (*shaded green*), the neutrino ‘floor’ (*shaded yellow*) and the prospective sensitivity of the LZ experiment (*purple*) [137]

We display as a black line in Fig. 20 the profile likelihood in the pMSSM10 for the ratio of $\mathrm{BR}(B_s \rightarrow \mu ^+\mu ^-)$ to the SM value. This can be compared with the $\chi ^2$ penalty from the experimental constraint on $\mathrm{BR}(B_s \rightarrow \mu ^+\mu ^-)$, which is shown as a red line. It is interesting to note that in the pMSSM10 both enhancement and suppression are possible, as opposed to the CMSSM, the NUHM1 and the NUHM2 [53, 59], in which a suppression was not possible and only an enhancement was allowed. This comes about because the extra parameters in the pMSSM10 make possible some negative interference between the SM and SUSY amplitudes, which is not possible in the other models when the various other constraints are implemented.

### Direct dark matter detection

The left panel of Fig. 21 displays the one-dimensional profile likelihood in the pMSSM10 for $m_{\tilde{\chi }^0_{1}}$ with the same colour coding as in Fig. 13. We see that, in contrast to the other models, the pMSSM10 favours a low mass for the $\tilde{\chi }^0_{1}$, driven again by the $(g-2)_\mu$ constraint. The right panel of Fig. 21 displays the two-dimensional profile likelihood for the lightest neutralino mass versus the spin-independent cross section, where the red and blue contours show the 68 and 95 % CL levels, respectively. The region that is excluded by LUX [92] and XENON100 [93] is shaded green, whereas the ‘floor’ below which the background from atmospheric neutrinos dominates is shaded yellow [137]. The low-mass vertical 95 % CL strips are due to points where the relic LSP density is brought into the cosmological range by annihilations through direct-channel *Z* and *h* poles.

**Fig. 22:**
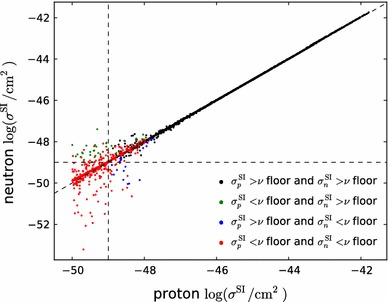
Scatter plot of the cross sections for spin-independent scattering on a proton target (*horizontal axis*) and on a neutron target (*vertical axis*) obtained from a sampling of pMSSM10 points within the 95 % CL region. The *diagonal dashed line* corresponds to equal spin-independent cross sections on proton and neutron targets. The *colour coding* distinguishes between points with either $\sigma ^\mathrm{SI}_p$ and/or $\sigma ^\mathrm{SI}_n$ above or below the neutino ‘floor’ seen in Fig. 21

It is interesting to note that the pMSSM10 fit prefers rather high values of the spin-independent cross section after application of the LHC8 constraints: lower values could be reached for a Bino-like LSP, but the dark matter density constraint would then require stau coannihilation and *t*-channel slepton exchange, which are, however, disfavoured by the combined effects of the $$\mathrm{LHC8}_\mathrm{EWK}$$ and $$(g-2)_\mu $$ constraints. Our best-fit region is close to the present experimental upper limit on $$\sigma ^\mathrm{SI}_p$$ [92], and consequently within reach of future direct detection experiments such as LZ [137], as indicated by the magenta line in the right panel of Fig. 21. On the other hand, we note that before applying these constraints, and even afterwards at the 95 % CL, there are values for $$\sigma ^\mathrm{SI}_p$$ that go far below this neutrino ‘floor’, highlighting the complementarity of direct detection experiments and searches at the LHC. Since these very low values of $$\sigma ^\mathrm{SI}_p$$ are due to cancellations between different contributions to the spin-independent scattering matrix element [138–142], one may ask whether the spin-independent cross sections on proton and neutron targets could be very different when this cancellation occurs [143, 144]. More specifically, one may wonder whether, for models in which $$\sigma ^\mathrm{SI}_p$$ is below the neutrino ‘floor’, the cross section $$\sigma ^\mathrm{SI}_n$$ for scattering on a neutron target may be less suppressed, perhaps remaining above the neutrino ‘floor’? As we see in Fig. 22, the spin-independent cross sections on proton and neutron targets are generally very similar when $$\sigma ^\mathrm{SI}_p$$$$> 10^{-47}$$ cm$$^2$$, but may indeed be quite different when $$\sigma ^\mathrm{SI}_p$$$$< 10^{-49}$$ cm$$^2$$, which is approximately the lowest level of the neutrino ‘floor’, whose height varies as seen in the right panel of Fig. 21. Points coloured black (green) [blue] {red} have both $$\sigma ^\mathrm{SI}_p$$ and $$\sigma ^\mathrm{SI}_n$$ above the neutrino ‘floor’ shown in the right panel of Fig. 21 ($$\sigma ^\mathrm{SI}_p$$ below and $$\sigma ^\mathrm{SI}_n$$ above) [$$\sigma ^\mathrm{SI}_p$$ above and $$\sigma ^\mathrm{SI}_n$$ below] {$$\sigma ^\mathrm{SI}_p$$ and $$\sigma ^\mathrm{SI}_n$$ both below}. We see that there is a significant population of models whose spin-independent scattering cross sections on protons and neutrons are both below the ‘floor’ (indicated in red), so there is no ‘no-lose’ theorem for dark matter scattering in the pMSSM10.^13^

## Extrapolation to high scales

In our analysis of the pMSSM10 we have not imposed any restriction on the possible extrapolation of the (purely phenomenological) soft SUSY-breaking parameters to high scales using the renormalisation-group equations. In many cases, one could expect that renormalisation by the gaugino masses may drive some soft supersymmetry-breaking sfermion masses-squared $m_0^2$ to negative values at high-energy scales [147]. This raises cosmological issues that have been studied, for example, in [148], and such scenarios do not necessarily lead to an unacceptable evolution of the Universe. However, it is interesting to study the implications of requiring $m_0^2 >0$. We emphasise that this cut reduces the data set significantly, and one may anticipate that part of the parameter space would be recovered in a dedicated scan. Nevertheless, we expect that the main features discussed here would be present also in a more complete scan.

**Fig. 23:**
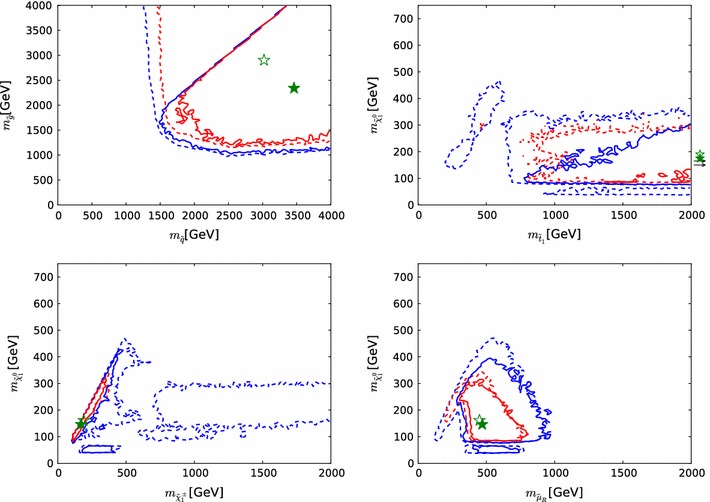
The impacts of the optional anti-tachyon cut on the two-dimensional profile likelihood functions in the $(m_{\tilde{q}}, m_{\tilde{g}})$, $(m_{\tilde{t}_{1}}, m_{\tilde{\chi }^0_{1}})$, $(m_{\tilde{\chi }^\pm _{1}}, m_{\tilde{\chi }^0_{1}})$ and $(m_{\tilde{\mu }_{R}}, m_{\tilde{\chi }^0_{1}})$ planes. In each panel the *solid (dashed) red/blue contours* denote the $\Delta \chi ^2 = 2.30/5.99$ level contours for the case where we do (not) apply the anti-tachyon constraint, respectively. The *green filled* and *empty stars* indicate the corresponding best-fit points

Figure 23 displays the two-dimensional likelihood functions in some relevant sparticle mass planes. In each panel, the red (blue) lines are the 68 % (95 %) CL contours, the solid (dashed) lines being after (before) a cut requiring $m_0^2 > 0$ for all the sleptons and squarks at the GUT scale $\sim 2\cdot 10^{16} \,\, \mathrm {GeV}$. The upper left panel shows the $(m_{\tilde{q}}, m_{\tilde{g}})$ plane, and they can be compared with Fig. 3 (upper left plot). We see that the primary impact of the anti-tachyon cut is to remove all models above a diagonal line where the negative renormalisation by $M_3$ drives the squark masses-squared negative at the GUT scale. The upper right panel of Fig. 23 shows the impact of the anti-tachyon cut on the $(m_{\tilde{t}_{1}}, m_{\tilde{\chi }^0_{1}})$ plane, which can be compared with the middle left panel of Fig. 10. Here the most obvious impact is to remove the compressed stop region where $m_{\tilde{t}_{1}} - m_{\tilde{\chi }^0_{1}}<m_t$.^14^ The lower left panel of Fig. 23 shows the impact in the $(m_{\tilde{\chi }^\pm _{1}}, m_{\tilde{\chi }^0_{1}})$ plane, where we see that many models with small $m_{\tilde{\chi }^\pm _{1}} - m_{\tilde{\chi }^0_{1}}$ survive the anti-tachyon cut. The anti-tachyon cut leads to a more pronounced preference for small values of $m_{\tilde{\chi }^0_{1}}$ and in particular $m_{\tilde{\chi }^\pm _{1}}$. Finally, the lower right panel of Fig. 23 displays the $(m_{\tilde{\mu }_{R}}, m_{\tilde{\chi }^0_{1}})$ plane, where we see that the anti-tachyon cut has very little effect, except to remove some points with small $m_{\tilde{\mu }_{R}} - m_{\tilde{\chi }^0_{1}}$. In particular, the best-fit values of $m_{\tilde{\mu }_{R}}$ and $m_{\tilde{\chi }^0_{1}}$ are little changed, and the mitigation of the $(g-2)_\mu$ anomaly in the pMSSM10 survives the anti-tachyon cut. However, we repeat that this cut may even not be necessary [148].

**Fig. 24:**
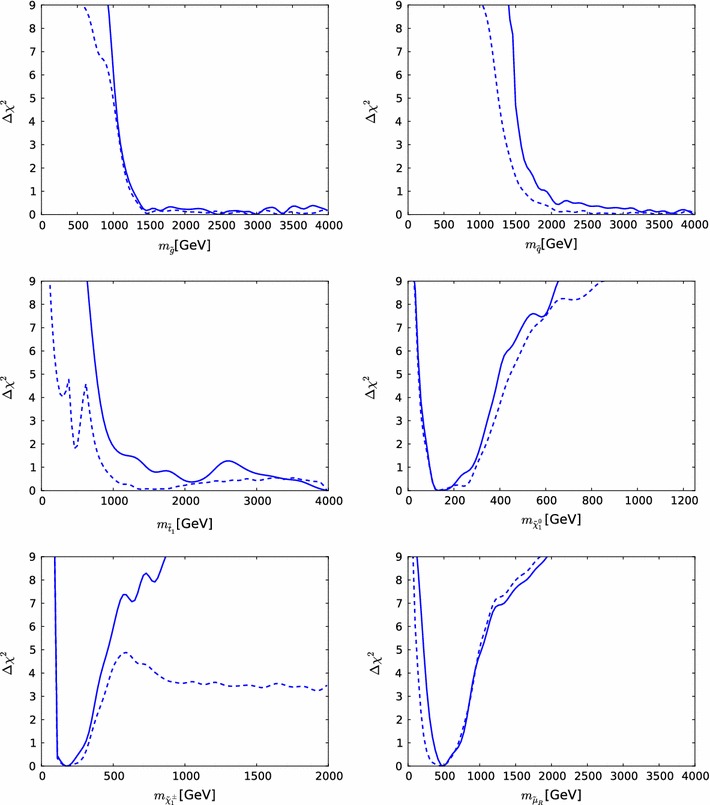
The impacts of the optional anti-tachyon cut on the one-dimensional profile likelihood functions for $m_{\tilde{g}}$, $m_{\tilde{q}}$, $m_{\tilde{t}_{1}}$, $m_{\tilde{\chi }^0_{1}}$, $m_{\tilde{\chi }^\pm _{1}}$ and $m_{\tilde{\mu }_{R}}$. In *each panel* the *solid* (*dashed*) *lines* are for the cases where we do (not) apply the anti-tachyon constraint, respectively

Figure 24 shows the impacts of the optional anti-tachyon cut on the one-dimensional profile likelihood functions for $$m_{\tilde{g}}$$, $$m_{\tilde{q}}$$, $$m_{\tilde{t}_{1}}$$, $$m_{\tilde{\chi }^0_{1}}$$, $$m_{\tilde{\chi }^\pm _{1}}$$ and $$m_{\tilde{\mu }_{R}}$$ (from top left to bottom right). We see that the $$\chi ^2$$ function for the gluino mass is little affected, whereas points with low $$m_{\tilde{q}}$$ are systematically removed, as one might expect from enforcing $$m_0^2 > 0$$. These effects can also be seen in the upper left panel of Fig. 23. As one would expect from the upper right panel of Fig. 23, points with low $$m_{\tilde{t}_{1}}$$ are also removed by the anti-tachyon cut, and the best-fit value of $$m_{\tilde{t}_{1}}$$ is increased by $$\sim $$$$1 \,\, \mathrm {TeV}$$. As seen in the middle right panel of Fig. 24, the one-dimensional likelihood function for $$m_{\tilde{\chi }^0_{1}}$$ is little affected, whereas that for $$m_{\tilde{\chi }^\pm _{1}}$$ is squeezed strongly. These effects reflect the behaviour in the $$(m_{\tilde{\chi }^\pm _{1}}, m_{\tilde{\chi }^0_{1}})$$ plane seen in the lower left panel of Fig. 23, where the favoured points lie in a narrow $$\tilde{\chi }^\pm _{1} - \tilde{\chi }^0_{1}$$ coannihilation strip. These points have $$M_1 \simeq M_2$$ at the electroweak scale, leading to the potential observability of neutralino/chargino pair production at an $$e^+e^-$$ collider with a centre-of-mass energy below $$1000 \,\, \mathrm {GeV}$$, as we discuss later. Finally, we see in the bottom right panel of Fig. 24 that the likelihood function for $$m_{\tilde{\mu }_{R}}$$ is little affected by the anti-tachyon cut, apart from the removal of some low-mass points as seen already in the lower right panel of Fig. 23. However, as already commented, the removal of these points does not prevent the pMSSM10 from addressing successfully the $$(g-2)_\mu $$ problem.

**Fig. 25:**
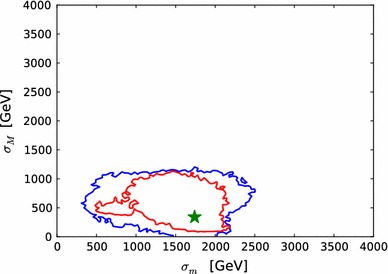
Two-dimensional likelihood function in the plane of the root-mean-square deviations from sfermion- and gaugino-mass universality, $\sigma _m$ and $\sigma _M$, defined in the text

As a final topic in this section, we discuss the departures from universality of the soft supersymmetry-breaking parameters in the sample that would survive the anti-tachyon cut. Figure 25 shows a plane of the root-mean-squared deviations from gaugino- and sfermion-mass universality, defined by

4 $$\begin{aligned} \sigma _{M, m} \equiv \sqrt{\sum _{i}^N (m_i - \bar{m})^2/N}, \end{aligned}$$

where the $$m_i$$ denote, respectively, the various gaugino-mass parameters and the square roots of the (positive) squark and slepton $$m^2_0$$ parameters in the pMSSM10 at the GUT scale, and $$\bar{m}$$ denotes their respective averages. Exact unification of the gaugino (sfermion) masses is achieved when $$\sigma _M$$ ($$\sigma _m$$) vanishes. We see that sfermion-mass universality is quite strongly violated, and gaugino-mass universality is also disfavoured, though still possible at the 95 % CL. As we have already commented, the favoured points in the narrow $$\tilde{\chi }^\pm _{1} $$–$$ \tilde{\chi }^0_{1}$$ coannihilation strip must have near-degenerate $$\tilde{\chi }^0_{1}$$ and $$\tilde{\chi }^0_{2}$$ and hence $$M_2 \simeq M_1$$ at the SUSY-breaking scale, corresponding to a breakdown of universality by a factor $$\sim $$2 at the GUT scale, i.e. $$M_1(M_\mathrm{GUT}) \sim 2 M_2(M_\mathrm{GUT})$$. As can also be inferred by comparing the top left and middle right panels of Fig. 24, a violation of GUT-scale $$M_3 $$–$$ M_1$$ universality is also suggested. Thus, refined future fits based on more data might lead to a preference for some different scenario for unification.

**Fig. 26:**
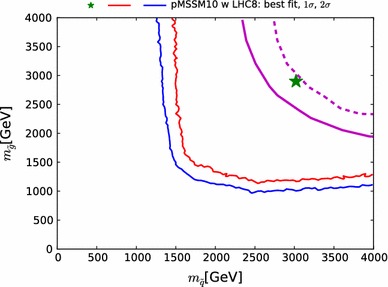
The $(m_{\tilde{q}}, m_{\tilde{g}})$ plane with our 68 and 95 % CL contours shown as *solid red* and *blue lines*, respectively, and the best-fit point as a *green star*. Also shown as *solid* (*dashed*) *magenta lines* are the estimated ATLAS sensitivities for 5-$\sigma$ discovery (95 % CL exclusion) of SUSY via the generic  search with 300 $\mathrm{fb}^{-1}$ at 14$\,\, \mathrm {TeV}$

## Prospects for sparticle detection in future LHC runs

At the time of writing, the LHC is starting run 2, taking data at $13 \,\, \mathrm {TeV}$, and it is expected that an integrated luminosity of 300 $\mathrm{fb}^{-1}$ will be collected by the early 2020s. There are also plans for a subsequent high-luminosity upgrade to accumulate 3000 $\mathrm{fb}^{-1}$. In this section we describe some prospects for future direct LHC searches for sparticles by ATLAS and CMS that follow from our analysis of the pMSSM10.

With the increase of the LHC centre-of-mass energy from 8 to 13 TeV for run 2, there will be large increases in the reaches for high-mass sparticle states. As shown in Fig 10, gluino masses $$\sim $$$$1.25$$ TeV (top left panel) and first- and second-generation squark masses $$\sim $$$$1.5$$ TeV (top right panel) are within our 68 % CL region. These masses will be probed by ATLAS and CMS with just a few $$\mathrm{fb}^{-1}$$ of data, demonstrating that already in an early phase of run 2 the discovery of SUSY might well be possible. For third-generation squarks, it is important to point out that besides masses of $$\sim $$$$800$$ GeV for $$\tilde{t}_{1}$$ (middle left panel) and $$\sim $$$$1$$ TeV for sbottoms (middle left panel), we also find in our 95 % CL region masses that are $$\sim $$$$200$$ to 600 GeV in the compressed stop region and $$\sim $$$$500$$ GeV for sbottoms. These regions have not been excluded by the LHC searches so far, but should become partly accessible in the first years of 13-TeV operation. As we comment later, in the cases of compressed spectrum charginos (bottom left panel) and sleptons (bottom right panel) comprehensive coverage of the preferred parameter space in the pMSSM10 by the LHC experiments will be challenging. However, depending on the decay modes of the electroweakly produced sparticles, early discovery at 13 TeV might also be possible.

Turning to the long-term prospects for the LHC, the ATLAS Collaboration has made physics studies that explore the discovery and exclusion reach of ATLAS with 300 and 3000 $\mathrm{fb}^{-1}$ at 14 TeV: see Fig. 13 of [149]. In Fig. 26 we display in the $(m_{\tilde{q}}, m_{\tilde{g}})$ plane our 68 % (95 %) CL contours in red (blue) as well as the estimated 5-$\sigma$ discovery (95 % $\mathrm{CL}_s$ exclusion) sensitivity with 300 $\mathrm{fb}^{-1}$ as solid (dashed) magenta contours.^15^ This shows that a substantial region of our preferred parameter space, including our best-fit point, is within reach of future LHC runs. However, we recall that the position of our best-fit point in the $(m_{\tilde{q}}, m_{\tilde{g}})$ plane is rather poorly determined.

In the following we revisit the mass planes of Fig. 10, assessing carefully the decay modes of the respective SUSY particles. A recurring theme is that the $\tilde{\chi }^\pm _{1}$ and $\tilde{\chi }^0_{2}$ are nearly degenerate in mass with $\tilde{\chi }^0_{1}$ in the 68 % CL region, so that squarks and sleptons decay via $\tilde{\chi }^\pm _{1}$ or $\tilde{\chi }^0_{2}$ in large fractions of the preferred parameter space. This general scenario is consistently indicated using pale blue shading.

**Fig. 27:**
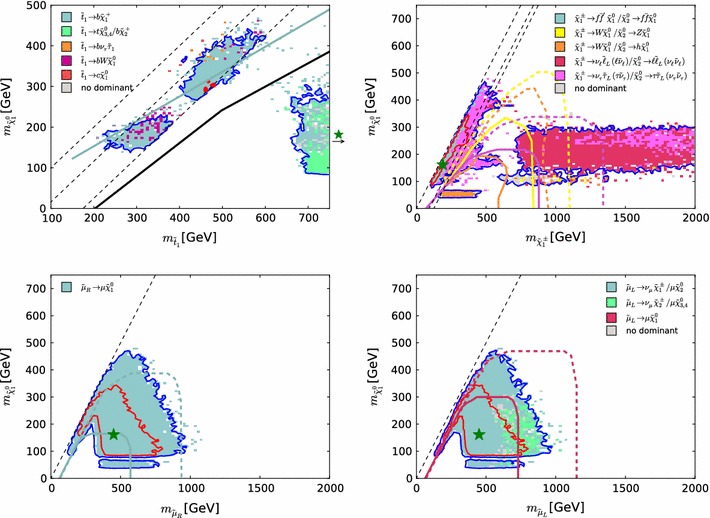
*Upper left panel* The $(m_{\tilde{t}_{1}}, m_{\tilde{\chi }^0_{1}})$ plane with our 68 and 95 % CL contours shown as *solid red* and *blue lines*, respectively, as well as coloured regions where the indicated branching ratios exceed 50 %. The projected LHC sensitivity with 300 $\mathrm{fb}^{-1}$ for $\tilde{t}_{1} \rightarrow \tilde{\chi }^0_{1} + t$ decays is shown as a *thick black line*, and the corresponding sensitivity for $\tilde{t}_{1} \rightarrow \tilde{\chi }^\pm _{1} b$ decays is shown as a *pale blue dashed line*. *Upper right panel* the $(m_{\tilde{\chi }^\pm _{1}}, m_{\tilde{\chi }^0_{1}})$ plane with our 68 and 95 % CL contours shown as *solid red and blue lines*, respectively. The *shadings* indicate where the branching ratios exceed 50 %. Also shown as *solid* (*dashed*) *yellow/orange/purple lines* are the projected LHC 95 % $\mathrm{CL}_s$ exclusion reaches for associated $\tilde{\chi }^\pm _{1}$ and $\tilde{\chi }^0_{2}$ production with decays via *W* / *Z*/*W* / *h*/${\tilde{\ell }_\mathrm{L}}/{\tilde{\nu }_{\ell _\mathrm{L}}}$/${\tilde{\tau }_\mathrm{L}}/{\tilde{\nu }_{\tau _\mathrm{L}}}$ with 300 (3000) $\mathrm{fb}^{-1}$ of data if these decays are dominant. *Lower left panel* the $(m_{\tilde{\mu }_{R}}, m_{\tilde{\chi }^0_{1}})$ plane with our 68 and 95 % CL contours shown as *solid red* and *blue lines*, respectively, with *pale blue shading* showing also where the branching ratio for $\tilde{\mu }_{R} \rightarrow \mu \tilde{\chi }^0_{1}$ is dominant, typically $\gtrsim 90~\%$. The *solid (dashed) pale blue lines* show our estimates of the LHC 95 % exclusion reach with 300 (3000) $\mathrm{fb}^{-1}$. *Lower right panel* similarly for the $(m_{\tilde{\mu }_{R}}, m_{\tilde{\chi }^0_{1}})$ plane, displaying the regions where the $\tilde{\mu }_{L} \rightarrow \mu \tilde{\chi }^0_{1}, \mu \tilde{\chi }^0_{2}/\nu _\mu \tilde{\chi }^\pm _{1}$ or $\mu \tilde{\chi }^0_{4}/\nu _\mu \tilde{\chi }^\pm _{2}$ decay modes have branching ratios exceeding 50 %. The *red lines* indicate the 95 % exclusion reach with 300 (3000) $\mathrm{fb}^{-1}$ if $\tilde{\mu }_{L} \rightarrow \mu \tilde{\chi }^0_{1}$ were dominant, but they are also indicative for the decay into $\tilde{\chi }^\pm _{1}/\tilde{\chi }^0_{2}$, which have masses nearly degenerate with $\tilde{\chi }^0_{1}$

With this in mind we turn to Fig. 27, where in the upper left panel we explore the possible future LHC sensitivity to direct stop production in the compressed-spectrum region. As previously, our present 68 % (95 %) CL contours are shown in red (blue). The colour shadings code the regions where the corresponding branching ratio, shown in the legend, exceeds 50 % for the point at each location that minimises the $\chi ^2$ function over the remaining parameters, and the thin diagonal dashed black lines correspond to $\Delta m \equiv m_{\tilde{t}_{1}} - m_{\tilde{\chi }^0_{1}} = 0, M_W + m_b$ and $m_t$. The solid dashed black lines show the projected LHC 95 % $\mathrm{CL}_s$ exclusion sensitivities for $\tilde{t}_{1} \rightarrow \tilde{\chi }^0_{1} t$ decays with 300 $\mathrm{fb}^{-1}$ [150] (similar sensitivity is found in this region with 3000 $\mathrm{fb}^{-1}$). These do not cover the case of a compressed-spectrum region, which includes the 95 % CL region where the dominant $\tilde{t}_{1}$ decays are to $\tilde{\chi }^\pm _{1} b$. Here we rescale from the present 95 % $\mathrm{CL}_s$ limit from the dibottom analysis, assuming that $m_{\tilde{\chi }^\pm _{1}} - m_{\tilde{\chi }^0_{1}} \sim 5 \,\, \mathrm {GeV}$ and using the Collider Reach tool [151] to rescale the production cross section, and assume that future LHC searches maintain the same search performance, i.e., the same signal yield after the event selection as present searches. We see that a search with 300 $\mathrm{fb}^{-1}$ of data (pale blue line) would already cover part of the 95 % CL region in the compressed-spectrum region, and the estimate for 3000 $\mathrm{fb}^{-1}$ is similar.

In the upper right panel of Fig. 27, we explore the possible future LHC sensitivity in the $(m_{\tilde{\chi }^\pm _{1}}, m_{\tilde{\chi }^0_{1}})$ plane, where our current 68 % (95 %) CL contours are again shown in red (blue) and the best-fit point is indicated by a green star, and the thin diagonal dashed black lines correspond to $\Delta m \equiv m_{\tilde{\chi }^\pm _{1}} - m_{\tilde{\chi }^0_{1}} = 0, M_Z$ and $M_h$. We find values of $\Delta m \lesssim 100 \,\, \mathrm {GeV}$ as well as small $\tilde{\chi }^0_{1} - \tilde{\chi }^0_{2}$ and $\tilde{\chi }^0_{2} - \tilde{\chi }^\pm _{1}$ mass differences in the region favoured at the 68 % CL. However, values of $\Delta m < 10 \,\, \mathrm {GeV}$ are disfavoured by $\Delta \chi ^2 \gtrsim 8$, so we do not expect long-lived particle and/or disappearing track signatures. We use colour coding to display points in the $(m_{\tilde{\chi }^\pm _{1}}, m_{\tilde{\chi }^0_{1}})$ plane where the following $\tilde{\chi }^\pm _{1}/ \tilde{\chi }^0_{2}$ decay modes have branching ratios $>$50 %: via virtual bosons $\tilde{\chi }^\pm _1\rightarrow f \bar{f}^\prime \tilde{\chi }^0_{1} / \tilde{\chi }^0_2 \rightarrow f \bar{f} \tilde{\chi }^0_{1}$ (pale blue), via on-shell bosons $\tilde{\chi }^\pm _1\rightarrow W \tilde{\chi }^0_1 / \tilde{\chi }^0_2 \rightarrow Z \tilde{\chi }^0_1$ (yellow) or $\tilde{\chi }^\pm _1\rightarrow W \tilde{\chi }^0_1 / \tilde{\chi }^0_2 \rightarrow h \tilde{\chi }^0_1$ (orange), via sleptons $\tilde{\chi }^\pm _1\rightarrow \nu _\ell \tilde{\ell }_\mathrm{L} (\ell \tilde{\nu }_\ell )/ \tilde{\chi }^0_2 \rightarrow \ell \tilde{\ell }_\mathrm{L} ( \nu _\ell \tilde{\nu }_\ell )$ where $(\ell = e, \mu )$ (red) and $\tilde{\chi }^\pm _1\rightarrow \nu _\tau \tilde{\tau }_\mathrm{L} (\tau \tilde{\nu }_\tau )/ \tilde{\chi }^0_2 \rightarrow \tau \tilde{\tau }_\mathrm{L} ( \nu _\tau \tilde{\nu }_\tau )$ (purple), whereas points with no branching ratios exceeding 50 % are coloured grey. The ATLAS Collaboration has made available projections of its sensitivities for some relevant searches for associated $\tilde{\chi }^\pm _{1}$ and $\tilde{\chi }^0_{2}$ production with 300 (3000) $\mathrm{fb}^{-1}$ of data [152], which are also shown in the upper right plane of Fig. 27 as solid (dashed) contours in the same colours as the relevant decay modes: yellow for $\tilde{\chi }^\pm _{1}\tilde{\chi }^0_{2}$ via *WZ*, orange for $\tilde{\chi }^\pm _{1}\tilde{\chi }^0_{2}$ via *Wh*, red for $\tilde{\chi }^\pm _{1}\tilde{\chi }^0_{2}$ via ${\tilde{\ell }_\mathrm{L}}/\tilde{\nu }_\ell$ where $(\ell = e, \mu )$, and purple for $\tilde{\chi }^\pm _{1}\tilde{\chi }^0_{2}$ via ${\tilde{\tau }_1}/\tilde{\nu }_\tau$. With 300 $\mathrm{fb}^{-1}$ of data the *Wh* search should already cover essentially all of the 95 % CL island with $m_{\tilde{\chi }^0_{1}} \lesssim 80 \,\, \mathrm {GeV}$, where these branching ratios exceed 50 %.^16^ However, even with 3000 $\mathrm{fb}^{-1}$ of data these searches would have limited impact on the other 95 % CL regions, since there the branching ratios for these decays are typically small. More importantly, they would have no impact on the 68 % CL region. The most relevant searches in the region with near-degenerate $\tilde{\chi }^\pm _{1}, \tilde{\chi }^0_{2}$ and $\tilde{\chi }^0_{1}$ would be in hadronic final states sensitive to $\tilde{\chi }^\pm _1\rightarrow f \bar{f}^\prime \tilde{\chi }^0_{1} / \tilde{\chi }^0_2 \rightarrow f \bar{f} \tilde{\chi }^0_{1}$ and $\tilde{\chi }^\pm _1\rightarrow \nu _\tau \tilde{\tau }_\mathrm{L} (\tau \tilde{\nu }_\tau )/ \tilde{\chi }^0_2 \rightarrow \tau \tilde{\tau }_\mathrm{L} ( \nu _\tau \tilde{\nu }_\tau )$. Searches for compressed charginos/neutralinos have been explored in [153], where some sensitivity was found up to $m_{\tilde{\chi }^\pm _{1}}/m_{\tilde{\chi }^0_{2}}\lesssim 300\,\, \mathrm {GeV}$ (after 3000 $\mathrm{fb}^{-1}$), although for very small mass differences $(m_{\tilde{\chi }^\pm _{1}} \simeq m_{\tilde{\chi }^0_{2}}) - m_{\tilde{\chi }^0_{1}}\lesssim 20\,\, \mathrm {GeV}$ and with optimistic assumptions concerning the possible systematic uncertainties.

**Fig. 28:**
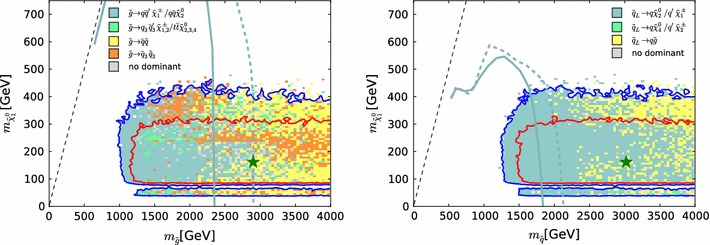
As in Fig. 27, but for ${\tilde{g}}$ decays (*left panel*) and ${\tilde{q}}$ decays (*right panel*). The *pale blue solid* (*dashed*) *lines* show the estimated sensitivities with 300 $\mathrm{fb}^{-1}$(3000 $\mathrm{fb}^{-1}$) for (*left panel*) ${\tilde{g}} \rightarrow q {\bar{q}}^\prime \tilde{\chi }^\pm _{1}, q {\bar{q}} \tilde{\chi }^0_{2}$ and (*right panel*) ${\tilde{q}_\mathrm{L}} \rightarrow q \tilde{\chi }^0_{2}, q^\prime \tilde{\chi }^\pm _{1}$

The lower left panel of Fig. 27 provides information as regards the dominant branching ratio for the $\tilde{\mu }_{R}$ in the favoured region of the $(m_{\tilde{\mu }_{R}}, m_{\tilde{\chi }^0_{1}})$ plane: as in the previous panels, our current 68 % (95 %) CL contours are in red (blue). We see that the branching ratio for $\tilde{\mu }_{R} \rightarrow \mu \tilde{\chi }^0_{1}$ exceeds 50 % in all of the 95 % CL region. We also show projections of the possible future sensitivities of the LHC with 300 (3000) $\mathrm{fb}^{-1}$ of data to $\tilde{\mu }_{R} \rightarrow \mu \tilde{\chi }^0_{1}$ decay as solid (dashed) pale blue lines. These projections were obtained via the following steps: (1) the present LHC 95 % $\mathrm{CL}_s$ limit for large $m_{\tilde{\mu }_{R}}/m_{\tilde{\chi }^0_{1}}$ was rescaled using the Collider Reach tool [151] to estimate the $\tilde{\mu }_{R}$ production cross section, and assuming that future LHC searches maintain the same search performance as present searches, and (2) we assumed that the shapes of the future sensitivity curves for other values of $m_{\tilde{\mu }_{R}}/m_{\tilde{\chi }^0_{1}}$ would be the same as for the current searches. We see that with 300 $\mathrm{fb}^{-1}$ the LHC would already explore a substantial part of the current 68 % CL region in the $(m_{\tilde{\mu }_{R}}, m_{\tilde{\chi }^0_{1}})$ plane, and that most of the 95 % CL region could be explored with 3000 $\mathrm{fb}^{-1}$ but missing a narrow band where $m_{\tilde{\mu }_\mathrm{r}} - m_{\tilde{\chi }^0_{1}}$ is small.

The favoured region of the $(m_{\tilde{\mu }_{L}}, m_{\tilde{\chi }^0_{1}})$ plane, shown in the lower right panel of Fig. 27, looks similar, but the dominant decay modes are more varied: any of the decays $\tilde{\mu }_{L} \rightarrow \mu \tilde{\chi }^0_{1},~\nu _\mu \tilde{\chi }^\pm _{1}/\mu \tilde{\chi }^0_{2}$ or $\nu _\mu \tilde{\chi }^\pm _{2}/\mu \tilde{\chi }^0_{4}$ have branching ratios exceeding 50 %. However, the $\tilde{\mu }_{L} \rightarrow \mu \tilde{\chi }^0_{1}$ decay mode dominates only when $m_{\tilde{\mu }_{L}} - m_{\tilde{\chi }^0_{1}}$ is very small. We have used the same approach as used above for projecting the $\tilde{\mu }_{R}$ sensitivity to the $\mu \tilde{\chi }^0_{1}$ decay mode also to estimate the future $\tilde{\mu }_{L}$ sensitivity, as shown by the solid (300 $\mathrm{fb}^{-1}$) and dashed (3000 $\mathrm{fb}^{-1}$) deep red lines. Although this projection assumes decays directly into $\tilde{\chi }^0_{1}$, it may have a similar sensitivity to the decay into $\tilde{\chi }^\pm _{1}/\tilde{\chi }^0_{2}$.

Figure 28 displays the corresponding $(m_{\tilde{g}}, m_{\tilde{\chi }^0_{1}})$ and $(m_{\tilde{q}}, m_{\tilde{\chi }^0_{1}})$ planes. In the ${\tilde{g}}$ case (left panel), we see that a number of different decay modes may have a branching ratio exceeding 50 %: via off-shell first- and second-generation squarks into charginos/neutralinos $\tilde{g}\rightarrow q \bar{q}^\prime \tilde{\chi }^\pm _1 / q \bar{q}\tilde{\chi }^0_2$ (pale blue), through off-shell third-generation squarks into (heavier) charginos/neutralinos $\tilde{g}\rightarrow q_3 \bar{q}_3^\prime \tilde{\chi }^\pm _{1,2} / t \bar{t} \tilde{\chi }^0_{2,3,4}$ (green), into first- and second-generation squarks $\tilde{g}\rightarrow \bar{q} \tilde{q}$ (yellow) or third-generation squarks $\tilde{g}\rightarrow \bar{q}_3 \tilde{q}_3$ (orange). However, none of these decay modes may have a branching ratio exceeding 50 % (grey). In the ${\tilde{q}}$ case (right panel), the decays of ${\tilde{q}}\rightarrow q^\prime \tilde{\chi }^\pm _{1}/q \tilde{\chi }^0_{2}$ (pale blue) are usually dominant, particularly at lower masses, though in some cases decays into gluinos $\tilde{q}_\mathrm{L}\rightarrow q\tilde{g}$ and at low $m_{\tilde{\chi }^0_{1}}$ decays into heavier neutralinos/charginos $\tilde{q}_\mathrm{L}\rightarrow q\tilde{\chi }^0_4 / q^\prime \tilde{\chi }^\pm _2$ (pale green) are dominant. The ATLAS Collaboration has presented projected exclusion limits for $\tilde{g} \rightarrow q \bar{q} \tilde{\chi }^0_{1}$ and $\tilde{q} \rightarrow q \tilde{\chi }^0_{1}$ simplified models with 300 (3000) fb$^{-1}$ of data at 14 TeV in [152]. Recalling that $m_{\tilde{\chi }^\pm _{1}} (m_{\tilde{\chi }^0_{2}}) \simeq m_{\tilde{\chi }^0_{1}}$ in our 68 % CL region, these simplified model limits may be applicable in the pale blue regions in Fig. 28 where $\tilde{g} \rightarrow q \bar{q}^\prime \tilde{\chi }^\pm _{1} / q \bar{q} \tilde{\chi }^0_{2}$ ($\tilde{q} \rightarrow q' \tilde{\chi }^\pm _{1} / q \tilde{\chi }^0_{2}$) dominate the gluino (squark) decay modes. We overlay these projected limits in Fig. 28. The solid (dashed) curves corresponds to the 300 (3000) fb$^{-1}$ data. In the ($m_{\tilde{g}}$, $m_{\tilde{\chi }^0_{1}}$) plane we can see that a large part of our 68 and 95 % CL regions can be probed with 300 (3000) fb$^{-1}$ data. Indeed, our best-fit point lies on the projected limit for 3000 fb$^{-1}$. We also see in the ($m_{\tilde{q}}$, $m_{\tilde{\chi }^0_{1}}$) plane that some parts of our 68 and 95 % CL regions can be explored with 300 (3000) fb$^{-1}$ data, although the projected limit presented in [152] with 3000 fb$^{-1}$ data does not reach our best-fit point.

**Fig. 29:**
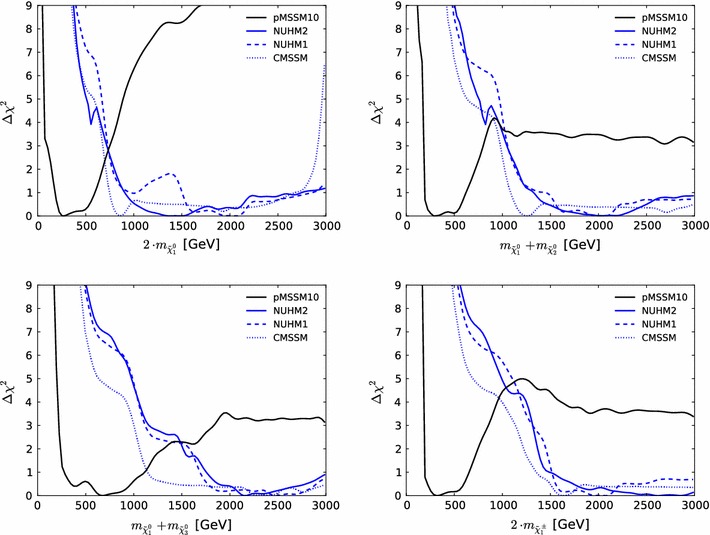
The one-dimensional profile likelihood functions for various thresholds in $e^+ e^-$ annihilation. *Upper left panel* the threshold for $\tilde{\chi }^0_{1} \tilde{\chi }^0_{1}$ production. *Upper right panel* the threshold for associated $\tilde{\chi }^0_{1} \tilde{\chi }^0_{2}$ production. *Lower left panel* the threshold for associated $\tilde{\chi }^0_{1} \tilde{\chi }^0_{3}$ production. *Lower right panel* the threshold for $\tilde{\chi }^\pm _{1} \tilde{\chi }^\mp _{1}$ production

Finally, we turn the prospects for discovery with 300 $\mathrm{fb}^{-1}$ of our benchmark points, starting with our global best-fit point (Fig. 11), which is just inside the reach of generic  searches (see Fig. 26), well in reach for the slepton searches (lower panels of Fig. 27), and even potentially within reach of the compressed-chargino/neutralino searches, as discussed in [153], due to its small mass splitting $m_{\tilde{\chi }^\pm _{1}}-m_{\tilde{\chi }^0_{2}}\simeq 20\,\, \mathrm {GeV}$.

As for our local best-fit point in the low-$m_{\tilde{t}_{1}}$ region (see the upper left panel of Fig. 14), it lies just within the reach of future searches in the compressed-stop region (upper left panel of Fig. 27), as well as slepton searches (lower panels of Fig. 27), but it would be difficult to access via chargino/neutralino searches, because of the low-mass splittings seen in Fig. 14 and the relatively high $m_{\tilde{\chi }^0_{1}}\simeq 300\,\, \mathrm {GeV}$. The relatively large contribution of the $\mathrm{LHC8}_\mathrm{col}$ constraint for this point, seen in the third row of Table 5, indicates that this point may be accessible via jets $+$*X*$+$ searches early in run 2.

In the cases of the low-$m_{\tilde{q}}$ and/or -$m_{\tilde{g}}$ points, by construction these points could also be discovered early in run 2 of the LHC, since they lie very close to the current 68 % CL boundary in the $(m_{\tilde{q}}, m_{\tilde{g}})$ plane shown in Fig. 26. This feature is also indicated by the significant contributions to the global $\chi ^2$ functions for these points that can also be seen the third row of Table 5.

## Prospects for sparticle detection at a future $e^+ e^-$ collider

Figure 29 displays the one-dimensional $\chi ^2$ functions for the lowest particle pair- and associated chargino and neutralino production thresholds in $e^+ e^-$ annihilation in the pMSSM10 (black), compared with their counterparts in the CMSSM (dotted blue), NUHM1 (dashed blue) and NUHM2 (solid blue). In the cases of $\tilde{\chi }^0_{1} \tilde{\chi }^0_{1}$ (upper left panel), $\tilde{\chi }^0_{1} \tilde{\chi }^0_{2}$ (upper right panel) and $\tilde{\chi }^\pm _{1} \tilde{\chi }^\mp _{1}$ (lower right panel) production, we see that the minima of the $\chi ^2$ functions in the pMSSM10 lie within reach of an $e^+ e^-$ collider with centre-of-mass energy 500 GeV, and that threshold locations favoured by $\Delta \chi ^2 \le 3$ would be within reach of a 1000 GeV collider, whereas no upper limit can be established at the 95 % CL. We also see that, in the case of $\tilde{\chi }^0_{1} \tilde{\chi }^0_{3}$ production (lower left panel) (which is very similar to the cases of $\tilde{\chi }^0_{1} \tilde{\chi }^0_{4}$, $\tilde{\chi }^0_{2} \tilde{\chi }^0_{3}$ and $\tilde{\chi }^\pm _{1} \tilde{\chi }^\mp _{2}$ production that we do not show) the minimum of the global $\chi ^2$ function for the threshold lies between 400 and 1000 GeV, again with no upper limit at the 95 % CL. It should be noted, however, that the optional anti-tachyon cut would indeed yield upper limits at the 95 % CL for those production modes. Referring back to the bottom right panel of Fig. 13 and the right panel of Fig. 16, we see that slepton pair-production thresholds may well also lie below 1000 GeV. In all cases, the expected locations of the thresholds in the CMSSM, NUHM1 and NUHM2 are at much higher centre-of-mass energies.

Thus, the accessibility of supersymmetric particles at $e^+e^-$ colliders is vastly different in the pMSSM10 and similar non-GUT models, as compared to the simplest GUT-based models.

## Conclusions

We have performed in this paper the first global likelihood analysis of the pMSSM using a frequentist approach that includes comprehensive treatments of the LHC8 constraints. This analysis required many developments and extensions of the MasterCode framework that are described in earlier sections of the paper. For example, in order to interpret the searches for coloured sparticles via jets $+$*X*$+$ signatures at LHC8, we combine searches sensitive to a variety of different cascade channels, whose relative probabilities depend on other model parameters. By combining a sufficiently complete set of channels [57], we capture essentially all the relevant decay channels, and so achieve a reliable $\mathrm{LHC8}_\mathrm{col}$ constraint. In the cases of the $\mathrm{LHC8}_\mathrm{EWK}$ constraints from searches for electroweak gauginos, Higgsinos and leptons, we constructed computationally efficient models for their contributions to the global likelihood function that mimic closely the more computationally intensive results from the Atom code. A similar procedure was used for the $\mathrm{LHC8}_\mathrm{stop}$ constraints from searches for models with compressed stop spectra, with the addition that we constructed the likelihoods for some simplified model searches using the Scorpion code. These procedures have all been validated extensively, as described in the text.

The results of our analysis of the pMSSM10 are described in Sect. 3, where we provide many details of the global likelihood function. We give there the parameters of our best-fit pMSSM10 point, while cautioning that its squark and gluino mass parameters are poorly constrained. On the other hand, some of the pMSSM10 parameters in the electroweak sector are relatively tightly constrained. For example, we find relatively narrow ranges of $\tilde{\chi }^0_{1}$ and slepton masses, which are quite light, and that $m_{\tilde{\chi }^0_{1}} \simeq m_{\tilde{\chi }^0_{2}} \simeq m_{\tilde{\chi }^\pm _{1}}$ in the region of parameter space that is preferred at the 68 % CL. The light spectrum of electroweakly interacting sparticles is preferred by the $(g-2)_\mu$ constraint, and the neutralino and chargino mass degeneracies are then required to obtain a satisfactory cold dark matter density. In addition to the best-fit point, we have presented and analyzed several alternative pMSSM10 points with low stop, squark and gluino masses that may serve as benchmarks for LHC run 2 analyses.^17^

One of the most striking features of our analysis is that the pMSSM10 can provide an excellent fit to $(g-2)_\mu$ while respecting all the LHC8 constraints, something that is not possible in models with universal soft supersymmetry-breaking terms at the GUT scale, such as the CMSSM, NUHM1 and NUHM2. A corollary is that there are interesting prospects for exploring the preferred region of the pMSSM10 parameter space in future experiments. For example, LHC searches at 14 TeV have excellent prospects for exploring the preferred regions of $m_{\tilde{q}}$ and $m_{\tilde{g}}$, as well as light ${\tilde{t}_1}$, ${\tilde{e}}$ and ${\tilde{\mu }}$ masses. Looking further ahead, the $(g-2)_\mu$-friendly regions of the pMSSM10 could be explored in detail with an $e^+ e^-$ collider operating at 500–1000 GeV in the centre of mass. In particular, such a machine would have a significant discovery potential in the preferred region for the lightest neutralino and chargino, while those states would be difficult to access at the LHC with the searches discussed in this paper. Also, we recall that the region of the pMSSM10 parameter space that is favoured at the 68 % CL after implementing the LHC8 constraints yields relatively large values of $\sigma ^\mathrm{SI}_p$ that should be accessible to forthcoming experiments: see the right panel of Fig. 21.

It is a characteristic of the pMSSM that the possibility of extrapolation to high renormalisation scales is not enforced, and indeed we find that most of our pMSSM10 parameter sets yield some tachyonic sfermion masses at high renormalisation scales. It is not clear that such models should be rejected out of hand [148], but it is reassuring that many features of our pMSSM10 fit would, nevertheless, be preserved if one required the absence of tachyons. On the other hand, the preferred region of the pMSSM10 parameter space has non-universal gaugino and sfermion masses. The former arise from the tension between $(g-2)_\mu$ (which favours small $M_{1, 2}$) and the $\mathrm{LHC8}_\mathrm{col}$ constraint (which favours larger $M_3$) as well as the dark matter constraint (which favours $M_1 \simeq M_2$ at the electroweak scale, not at the GUT scale). In parallel, sfermion-mass non-universality also arises from the tension between $(g-2)_\mu$ (which favours small $m_{\tilde{\mu }}$) and the $\mathrm{LHC8}_\mathrm{col}$ constraint (which favours large squark masses).

It would be desirable to extend our approach to more general variants of the pMSSM with fewer restrictions on the parameters. For example, it would be interesting to relax the assumption of a single slepton mass scale: this is unlikely to alter the preferred range of the ${\tilde{\mu }_{\mathrm{L,R}}}$, but would have important repercussions for dark matter density calculations. It would also be desirable to revisit in more general pMSSM scenarios the preferences we have found for neutralino and chargino mass degeneracies, and the constraints we find in the $(M_A, \tan \beta )$ plane, which are largely indirect (being due to the interplay between constraints whose combination may have different implications in more general pMSSM scenarios). However, we think that many features of our pMSSM10 analysis would persist in more general scenarios.

Finally, when interpreting the impacts of experimental searches in our preferred pMSSM10 region, it is important to take into account decay chains involving an intermediate chargino, which is required to be light in order to fulfil the relic density constraint. In a large part of our preferred parameter space the chargino is almost mass degenerate with $\tilde{\chi }^0_{1}$, and there are also regions with a sizeable mass difference that exhibit distinctive decay chains. Therefore, the pMSSM10 motivates interpreting searches not only in terms of the minimal decay chains of the simplified models presently being considered, but also with the $\tilde{\chi }^\pm _{1}$ (and possibly also the $\tilde{\chi }^0_{2}$) incorporated in the spectrum over a range of low masses.

We await with interest the verdict of future runs of the LHC.

## Footnotes

1 Scorpion was first developed by J. Marrouche, and developed further by O. Buchmueller, M. Citron, S. Malik and K.J. de Vries: details may be obtained by contacting O. Buchmueller.

2 Information about this code is available from K. A. Olive: it contains important contributions from T. Falk, A. Ferstl, G. Ganis, A. Mustafayev, J. McDonald, F. Luo, K. A. Olive, P. Sandick, Y. Santoso, V. Spanos, and M. Srednicki.

3 The corresponding ATLAS results [96] have a similar sensitivity, but they are documented less completely.

4 See footnote 1.

5 See footnote 1.

6 The other panels of Fig. 3 show the corresponding uncertainties in our treatments of the $\mathrm{LHC8}_\mathrm{EWK}$ and $\mathrm{LHC8}_\mathrm{stop}$ constraints, which are discussed later.

7 Here and in subsequent analogous parameter planes, we treat the $\Delta \chi ^2 = 2.30$ and 5.99 contours as proxies for the 68  and 95 % CL contours.

8 Our $\mathrm{LHC8}_\mathrm{EWK}$ and $\mathrm{LHC8}_\mathrm{stop}$ analyses described later also differ by less than one from the corresponding Scorpion/Atom evaluations. This is also true for the benchmark points introduced later.

9 However, the LEP SUSY constraints [124] are applied.

10 The striations in these bars reflect the non-monotonic behaviours of the $\chi ^2$ function visible in Fig. 13.

11 The $\chi ^2$ probabilities and values of $\chi ^2$ differ from those given in [53], as we have updated the CMSSM and NUHM analyses with the most recent Higgs mass determination [94] and other new information [10, 90]. We note, in particular, that the new BR($B_u \rightarrow \tau \nu _\tau$) measurement [90] improves the agreement with the SM and the SUSY models we study.

12 We do not show the corresponding results for the $\tilde{\mu }_{\mathrm{L}}$, which are very similar.

13 We do not include in the right panel of Fig. 21 and in Fig. 22 the contributions of loop-induced scattering off gluons [145]. In general, these contributions are relatively small [146], but they would also shift slightly the parameters of the models exhibiting strong cancellations in $\sigma ^\mathrm{SI}_p$ and $\sigma ^\mathrm{SI}_n$. We thank N. Nagata for discussions on these points.

14 The 68 % CL region extends to much larger values of $m_{\tilde{t}_{1}}$, where larger values of $m_{\tilde{\chi }^0_{1}}$ are also found.

15 The 5-$\sigma$ discovery contour for 3000 $\mathrm{fb}^{-1}$ is almost coincident with the 95 % exclusion contour for 300 $\mathrm{fb}^{-1}$.

16 However, it should be kept in mind that the projected exclusion regions always assume the relevant branching ratios to be equal to one, and real exclusion bounds with “mixed” branching ratios would in general be different.

17 SLHA files [86, 87] for these points can be downloaded from the MasterCode website [63].
